# Substrate-specific effects point to the important role of Y361 as part of the YER motif in closing the binding pocket of OCT1

**DOI:** 10.1016/j.jbc.2025.108318

**Published:** 2025-02-14

**Authors:** Sarah Römer, Erika Lazzarin, Anna Neumann, Erik Lindemann, Marleen J. Meyer-Tönnies, Thomas Stockner, Mladen V. Tzvetkov

**Affiliations:** 1 Department of General Pharmacology, Institute of Pharmacology, Center of Drug Absorption and Transport (C_DAT), University Medicine Greifswald, Greifswald, Germany; 2 Institute of Pharmacology, Center for Physiology and Pharmacology, Vienna, Austria

**Keywords:** YER motif, organic cation transporter 1, OCT1, *SLC22A1*, membrane transport, structure–function, transporter, drug transport

## Abstract

Organic cation transporter 1 (OCT1) is located in the sinusoidal membrane of human hepatocytes. It mediates the uptake of hydrophilic organic cationic drugs in hepatocytes and thus determine their systemic concentrations. OCT1 has a broad spectrum of structurally diverse substrates like metformin, sumatriptan, trospium, and fenoterol. Recent cryo-EM data suggested that Y361 (tyrosine_361_), E386 (glutamate_386_), and R439 (arginine_439_), referred to as the YER motif, could be important for transport. Building on this, we used extensive functional analyses to investigate the general function and the substrate-specific effects of the YER motif. We determined the activity of the Y361A, E386A, and R439A mutants for 15 OCT1 substrates. Extended mutagenesis revealed the negative charge of E386 and the positive charge of R439 as essential for the transport of all substrates tested. Charge reversal mutants, E386R–R439E, did not restore transport activity, suggesting that at least one of the two amino acids is involved in additional interactions essential for transport. Y361 exhibited substrate-specific effects. The Y361A mutant transported fenoterol but not pirbuterol or other beta_2_-adrenergic drugs with only one aromatic ring. Molecular dynamics simulations suggested that substrates with aromatic or lipophilic characteristics may compensate for the missing aromatic ring at position 361. Only tryptophan at codon 361 efficiently rescued the transport of the Y361A mutant supporting hydrogen bound interaction with E386 and R439. Our study confirms that the YER motif is essential for OCT1 transport and points to Y361 as a lever that interacts with E386 and R439 to trigger the closing of the binding pocket of human OCT1.

The human organic cation transporter 1 (OCT1) is expressed almost exclusively in the sinusoidal membrane of hepatocytes (1, 2, 3). OCT1 mediates the uptake of weakly basic or positively charged hydrophilic drugs like metformin, morphine, sumatriptan, and fenoterol from the bloodstream into hepatocytes. This may determine the hepatic concentrations of these drugs and be a rate-limiting step in their hepatic metabolism. Individuals with loss or reduction of OCT1 activity because of genetic polymorphisms showed altered systemic concentrations of fenoterol and sumatriptan (4, 5) and may have reduced hepatic concentration of metformin and proguanil (6, 7).

OCT1 is a polyspecific transporter. More than 200 substances have been identified as OCT1 substrates (8, 9, 10). Alongside metformin, fenoterol, sumatriptan, and proguanil, OCT1 substrates are drugs like ipratropium, methylnaltrexone, trospium and endogenous compounds like serotonin and thiamine (4, 5, 11, 12, 13, 14, 15, 16). The substrate spectrum of OCT1 shows a broad variability in the recognized chemical structures, ranging from structures with low molecular complexity like tetraethylammonium (TEA^+^), a typical OCT1 model substance, to highly complex structures, such as ipratropium or methylnaltrexone. Since its initial cloning in 1994 (17), the broad substrate specificity of OCT1 is the focus of research, but the exact mechanisms remain unknown.

The OCT1 protein is composed of 12 pseudosymmetrical transmembrane helices (TMHs) with N and C termini located intracellularly. OCT1 belongs to the major facilitator superfamily, allowing facilitated diffusion of substrates across membranes following the rocker-switch mechanism. Thereby, substrates bind in the outward-open conformation of the transporter, induce a conformational transition toward an outward-occluded state, followed by a further switch toward an inward-occluded state, with final intracellular release of the substrate in the inward-open conformation.

Multiple amino acids have been suggested to be involved in interactions with the substrate in the substrate-binding pocket of OCT1. These include not only the negatively charged D474, which was initially suggested to interact with the positive charge of the substrate (18), but also F159, W217, and R439 (19). However, most of the experiments were performed with only a limited number of model substrates and could therefore not provide a profile of substrate-specific interactions. Previously, our group used a comparison of the substrate spectrum followed by chimeric constructs between mouse and human OCT1 (hOCT1) to identify amino acids involved in substrate-specific interactions (20). We were able to identify two amino acids in TMH1, C36 and F32, that were responsible for the strong differences in the transport kinetics between mouse and hOCT1. These exclusively functional data pointed toward an important role of TMH1.

Recently, multiple OCT1 structures were resolved using cryo-EM, confirming the spatial arrangement of the TMHs, as well as the rocker-switch transport mechanism (21, 22, 23). Based on the structures, some of the authors suggested that glutamate_386_ (E386) is involved in the direct interaction with the charge of the substrate (21). However, subsequently resolved structures failed to confirm a direct interaction (22, 23). More importantly, it was suggested that E386 could form together with tyrosine_361_ (Y361) and arginine_439_ (R439), a transport-critical YER motif (Y361, E386, and R439; (23), Figure 1*A*). However, the available functional data to support the structure-based predictions were limited to only few substrates and could not provide information about the substrate-specific effects (23).

**Figure 1:**
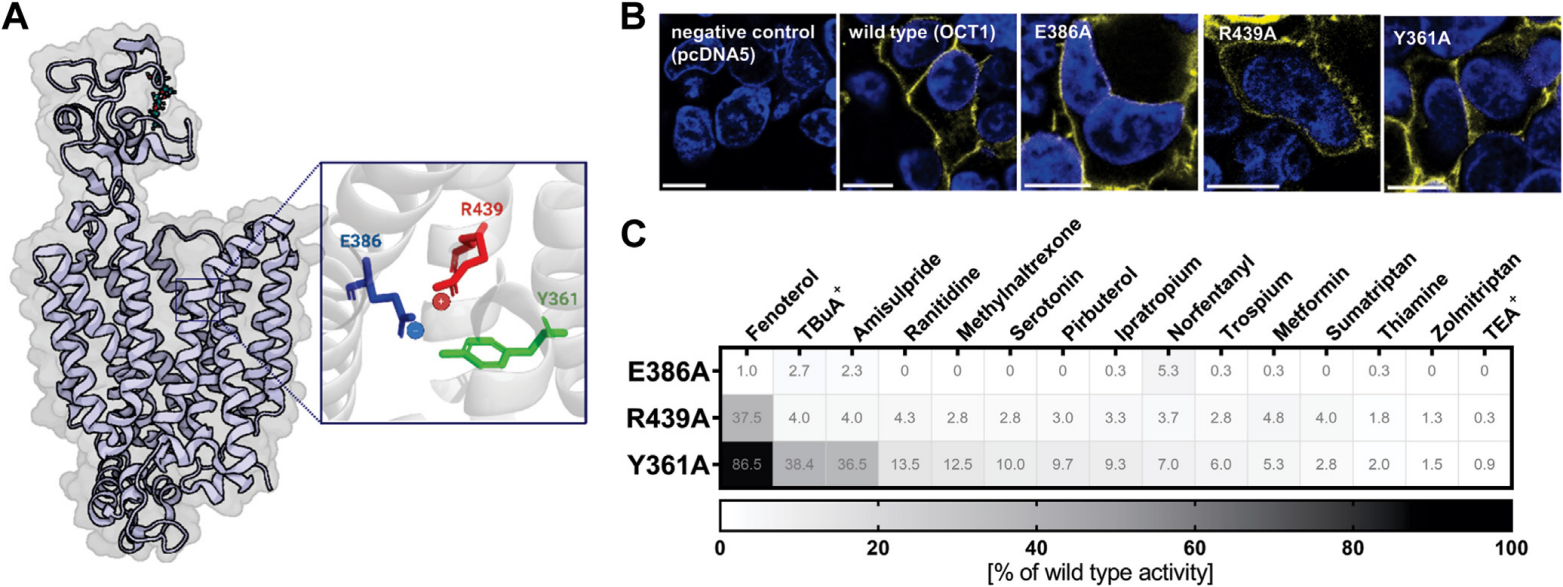
**Effects of single amino acids within the YER motif on OCT1-mediated transport**. *A*, schematic representation of the YER motif consisting of tyrosine_361_ (Y361, TMH 7), glutamate_386_ (E386, TMH 8), and arginine_439_ (R439, TMH 10) within the OCT1 structure. *B*, verification of membrane localization of alanine mutants in transfected HEK293 cells; scale bar represents 10 μm. *C*, uptake of known OCT1 substrates into HEK293 cells stably overexpressing the E386A, R439A, or Y361A mutant; active uptake was normalized to wildtype activity after subtraction of passive diffusion into empty vector control cells (pcDNA5); concentrations used are listed in Table S3; shown are means of n = 3 to 7 independent experiments. HEK293, human embryonic kidney 293 cell line; OCT1, organic cation transporter 1; TMH, transmembrane helix; YER motif, Y361, E386, and R439.

This study aimed to complement existing cryo-EM data with functional analyses to address substrate-specific effects of the YER motif and potential direct interactions with substrates. This will advance our understanding of the mechanism that allows OCT1 to recognize and transport a variety of chemical structures. Therefore, we generated human embryonic kidney 293 (HEK293) cells overexpressing mutants of E386, R439, and Y361 and analyzed their transport capabilities for a broad variety of OCT1 substrates. By applying ligand structure walking, followed by docking and molecular dynamics (MD) simulations, we then narrowed down the substrate moieties responsible for substrate-specific effects.

## Results

### Substrate-specific and substrate-unspecific effects of YER amino acids on OCT1-mediated transport

First, we mutated each of E386, R439, and Y361 to alanine. The mutants were stably transfected into HEK293 cells and analyzed for their membrane localization and transport capabilities by measuring the transport at a single concentration for 15 known OCT1 substrates (Fig. 1).

All 3 mutants were correctly localized in the cell membrane (Fig. 1*B*). Despite correct membrane localization, the E386A mutant was completely unable to transport any of the substrates tested (Fig. 1*C*). The R439A mutant was unable to transport all substrates except FNT, which retained 38% of the wildtype activity. In contrast to the E386A and R439A mutants, Y361A showed substantial transport of fenoterol, tetrabutylammonium (TBuA^+^), and amisulpride. Y361A transported not only fenoterol similarly to the wildtype (87% ± 11% of wildtype activity) but also TBuA^+^ (38% ± 6% of wildtype activity) and amisulpride (37% ± 3% of wildtype activity).

### The negative charge and the distance between the charge and the C-alpha atom of E386 are important for OCT1 transport

**Figure 2:**
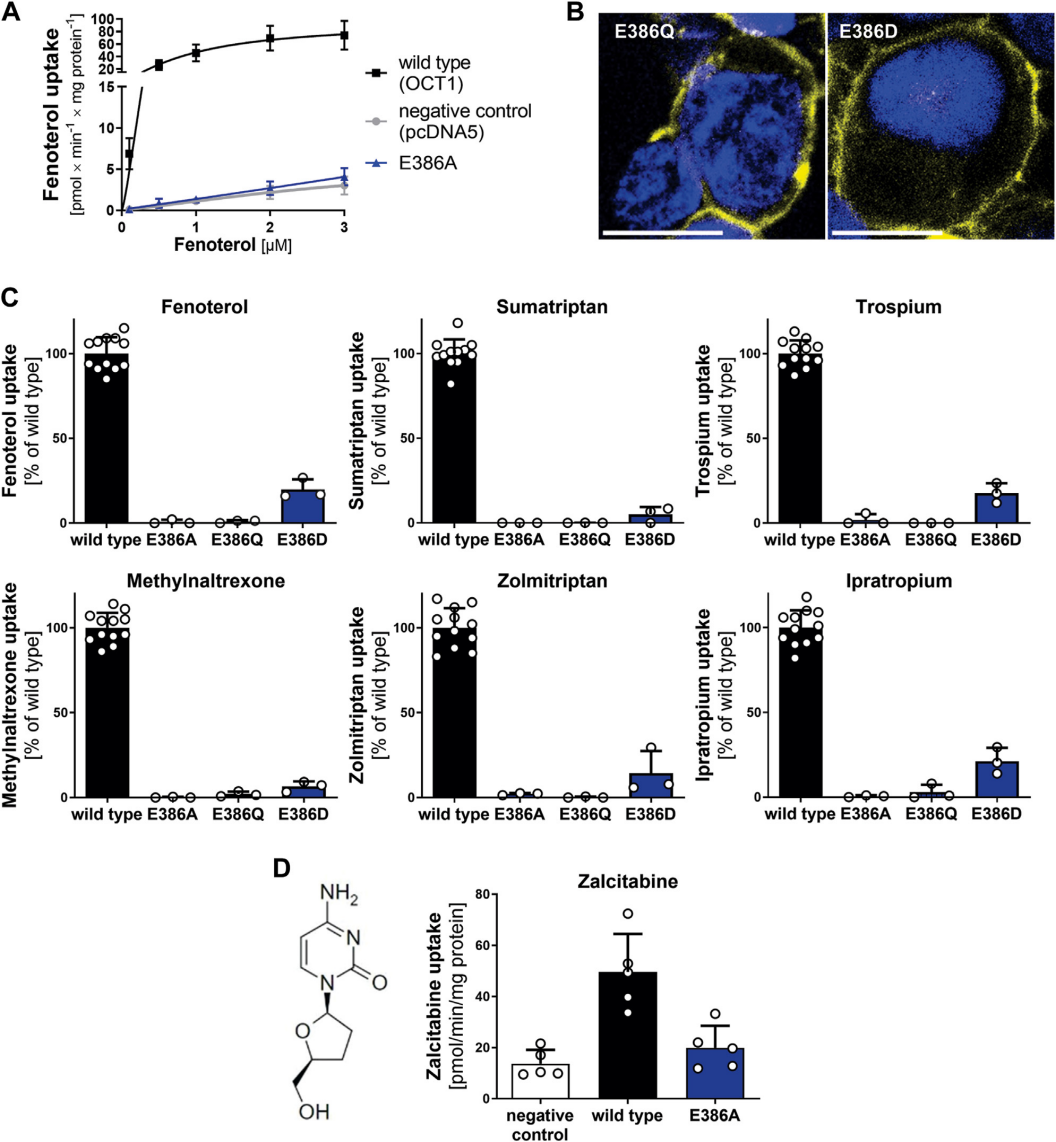
**Effects of E386 mutation on transport of multiple OCT1 substrates**. *A*, concentration-dependent uptake of fenoterol in HEK293 cells stably overexpressing the E386A mutant. *B*, verification of membrane localization of additional E386 mutants in transiently transfected HEK293 cells; scale bar represents 10 μm. *C*, uptake of key OCT1 substrates in HEK293 cells transiently transfected with E386 mutants; uptake was normalized on wildtype activity after subtraction of passive diffusion into empty vector control cells (pcDNA5). *D*, uptake of the uncharged substrate zalcitabine by the E386A mutant; concentrations used are listed in Table S3; shown are means ± SD of n = 3 to 5 independent experiments. HEK293, human embryonic kidney 293 cell line; OCT1, organic cation transporter 1.

As the E386A mutant lacked the ability to transport any substrate, we analyzed the role of E386 in more detail. The E386A mutant was able to transport neither fenoterol (Fig. 2*A*) nor sumatriptan or trospium (Fig. S1) over the entire concentration spectrum. This confirmed that E386 is essential for OCT1 transport.

Next, we analyzed whether the charge alone or other features of E386 are essential. By mutating glutamate to glutamine (E386Q), we removed the charge without altering the length and other properties of the side chain. Similarly to E386A, the E386Q mutant was correctly expressed (Fig. 2*B*) but lacked transport activity for all substrates tested (Fig. 2*C*). In the next experiment, we mutated not only the negatively charged amino acid glutamate to the shorter but also negatively charged aspartate (E386D). The E386D mutant was correctly localized in the plasma membrane (Fig. 2*B*) but could only partially retain transport function, reaching maximally 21% compared with wildtype activity (in case of ipratropium uptake; Fig. 2*C*). This suggests that the negative charge at position 386 is essential for transport and also its exact localization is important.

Next, we investigated whether the effects of the E386 mutation could be solely explained by the absence of an interaction partner for the positive charge of the substrate. To address this, we evaluated the impact of the E386A mutation on the transport of zalcitabine, a known OCT1 substrate that lacks a positive charge (10). Similar to all other substrates tested, the E386A mutant was unable to transport zalcitabine (Fig. 2*D*), suggesting that the role of E386 extends beyond a direct interaction with the positive charge of the substrate.

### The positive charge at position 439 is important for OCT1 transport

**Figure 3:**
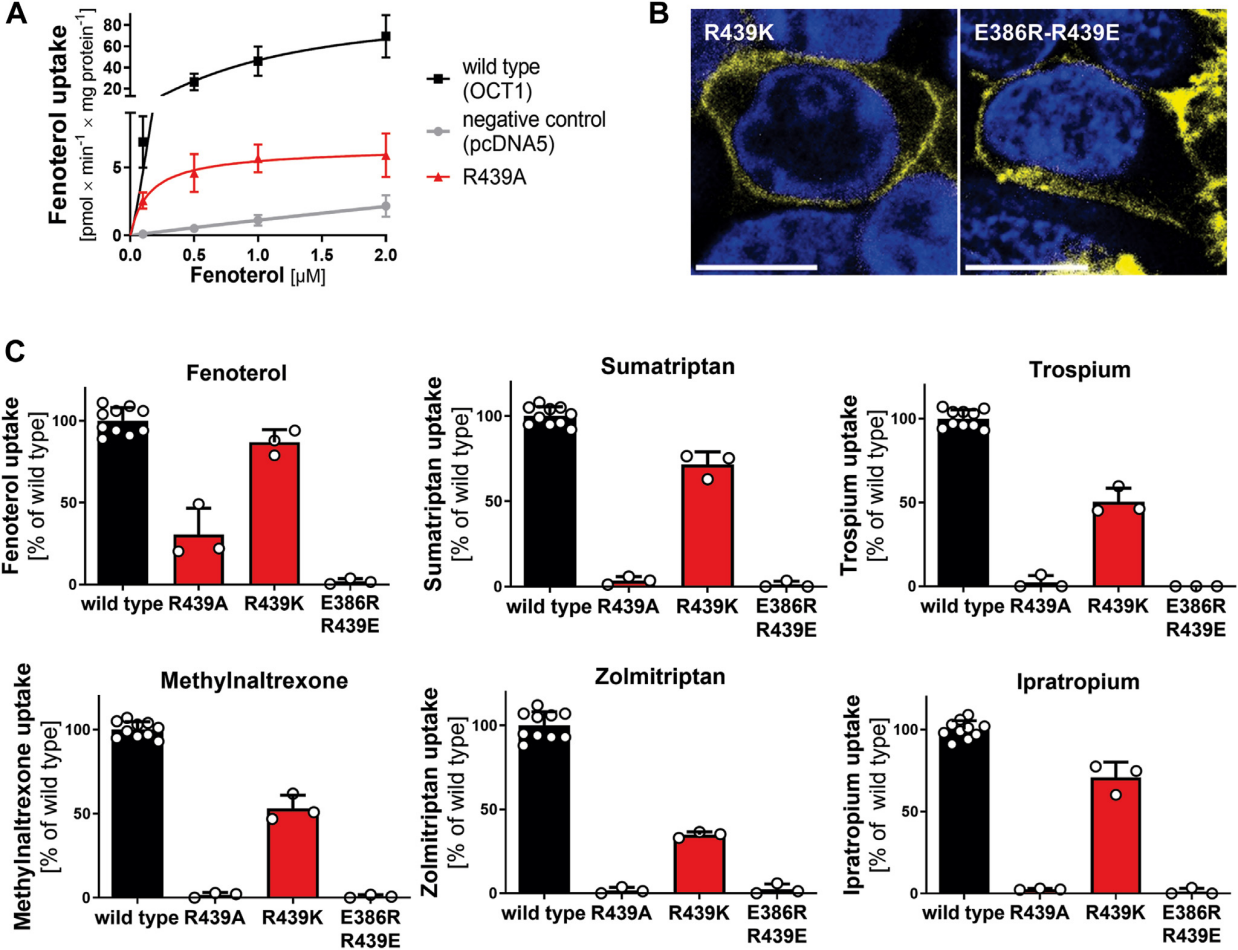
**Effects of R439 mutation on transport of multiple OCT1 substrates**. *A*, concentration-dependent uptake of fenoterol in HEK293 cells stably overexpressing the R439A mutant. *B*, verification of membrane localization of additional R439 mutants in transiently transfected HEK293 cells; scale bar 10 μm. *C*, uptake of key OCT1 substrates in HEK293 cells transiently transfected with the R439 mutants or the double mutant E386R-R439E; uptake was normalized on wildtype activity after subtraction of passive diffusion into empty vector control cells (pcDNA5); concentrations used are listed in Table S3. Shown are means ± SD of n = 3 independent experiments. HEK293, human embryonic kidney 293 cell line; OCT1, organic cation transporter 1.

Similar to E386, we initially assessed the ability of the R439A mutant to transport fenoterol across a broader concentration range. The R439A mutant partially retained the ability to transport fenoterol but not of any other substrate tested (Fig. 1*C*). However, its maximum transport velocity was reduced by 16-fold compared with wildtype, indicating an important role also of R439 for OCT1 transport (Fig. 3*A*).

Next, we analyzed whether the charge of R439 itself was important for the almost complete loss of function of the R439A mutant. When the positive charge was maintained by mutating arginine_439_ to lysine (R439K), transport activity was retained. The R439K mutant was correctly localized in the plasma membrane (Fig. 3*B*) and showed more than 50% of wildtype activity for most substrates (Fig. 3*C*). This indicates that a positively charged amino acid is the important feature for OCT1 function at position 439.

Cryo-EM structures showed distances of 3.9 to 7.1 Å between E386 and R439 that are sufficient to build a salt bridge between these two residues of the YER motif (21, 22, 23, 24). To prove whether the salt bridge alone is sufficient to explain the functional role of R439 and E386, we created a charge reversal double mutant by simultaneously mutating E386 and R439 (E386R–R439E). The double mutant was correctly localized in the plasma membrane (Fig. 3*B*) but was not able to transport any of the substrates tested (Fig. 3*C*). This suggests that the salt bridge interaction between E386 and R439 alone is not sufficient, and additional interactions of one or both of the partners with additional amino acids are of relevance for transport.

### Fenoterol and TBuA^+^ are substrates that rescued Y361A activity

**Figure 4:**
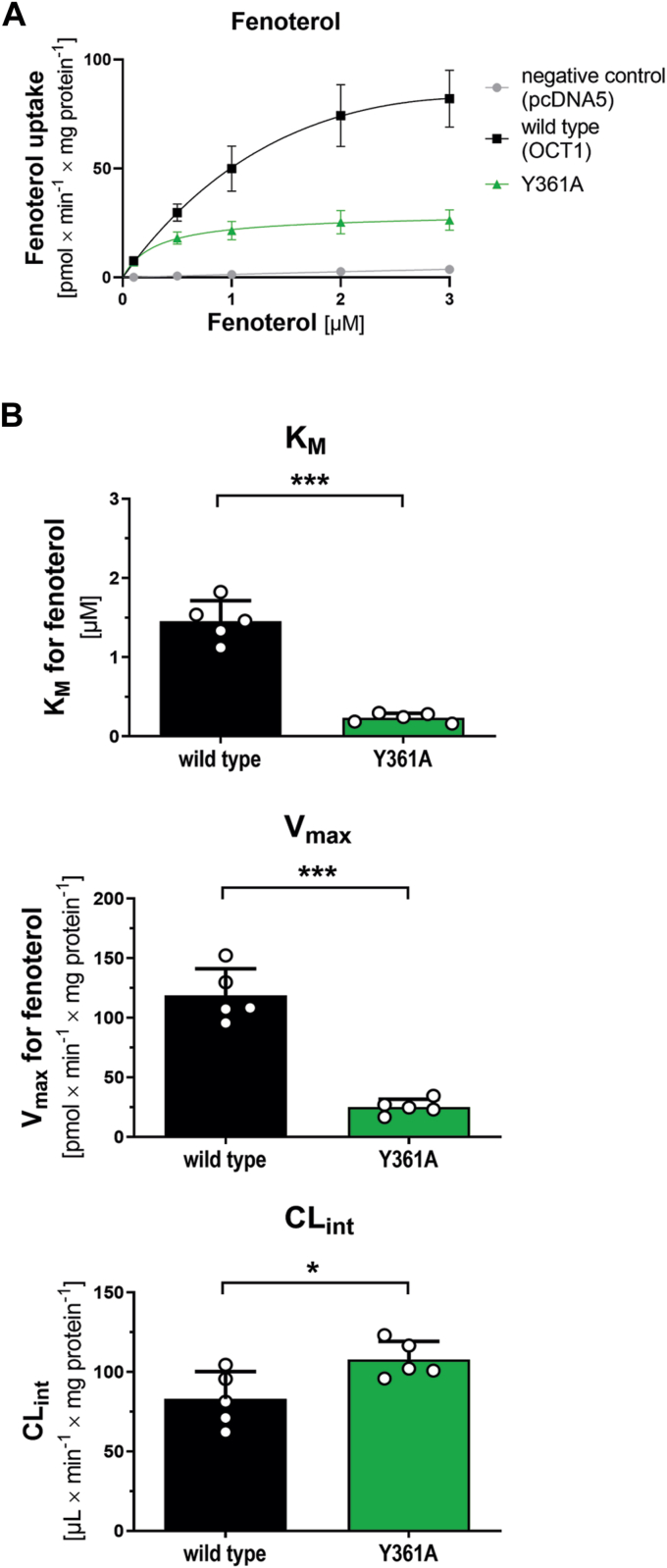
**Effects of Y361A mutation on fenoterol uptake**. *A*, concentration-dependent uptake of fenoterol in HEK cells stably overexpressing the Y361A mutant or wildtype OCT1; empty vector control cells (pcDNA5) were used as reference for passive diffusion. *B*, resulting pharmacokinetic parameters; two-tailed paired *t* test (∗*p* < 0.05, ∗∗∗*p* < 0.001); shown are means ± SD of n = 5 independent experiments. HEK293, human embryonic kidney 293 cell line; OCT1, organic cation transporter 1.

As the Y361A mutant demonstrated no substantial loss in activity when fenoterol was used as a substrate (Fig. 1), we further characterized the transport kinetics of fenoterol in the Y361A mutant (Fig. 4). The transport velocity (*v*_max_) of fenoterol was reduced by 4.7-fold (*p* = 0.0003, Fig. 4*B*) in the Y361A mutant compared with wildtype, whereas the affinity (*K*_*M*_) was increased by 6.2-fold (lower *K*_*M*_ value, *p* = 0.0002, Fig. 4*B*). However, because of the proportional decrease in both *v*_max_ and *K*_*M*_, the intrinsic clearance (CL_int_) for fenoterol remained largely unchanged between the Y361A and wildtype (1.3-fold decrease, *p* = 0.02) (Fig. 4*B*).

**Figure 5:**
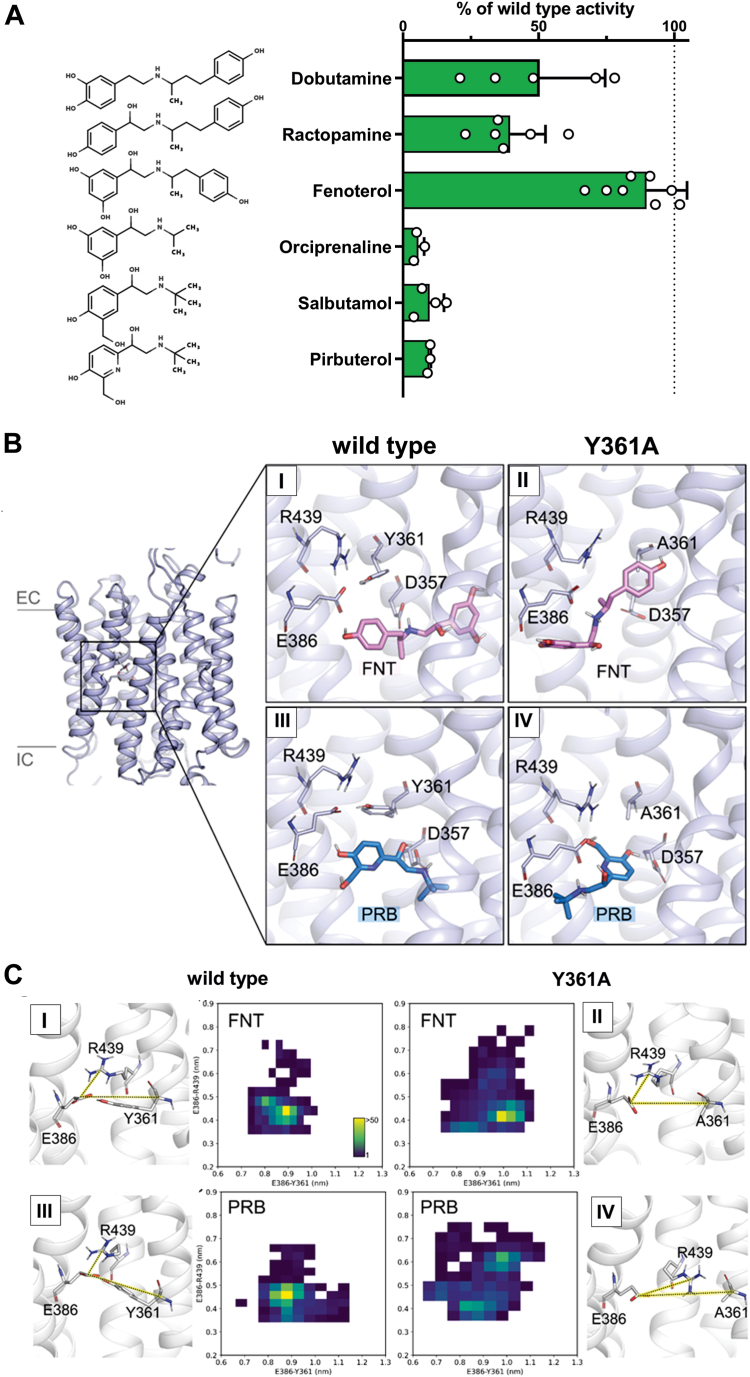
**The second ring of the beta_2_**-**adrenergic agonists is essential for rescuing Y361A activity**. *A*, influence of ligand structure of beta_2_ agonists on the role of Y361 in OCT1 transport; uptake of different beta_2_ agonists was measured in HEK293 cells stably overexpressing the Y361A mutant; active uptake was normalized on wildtype activity; shown are means ± SD of n = 3 to 8 independent experiments. *B*, compounds fenoterol (FNT; I, II) and pirbuterol (PRB; III, IV) were docked into the inward-occluded binding pocket of wildtype OCT1 (I, III) and Y361A mutant (II, IV), using the OCT1-verapamil bound cryo-EM structure (Protein Data Bank ID: 8ET8, (21)); resulting docking poses served as starting points for 6 unbiased 100 ns molecular dynamics simulations; trajectories were ranked based on MMPBSA free-energy calculations (Fig. S4), and the best end-pose for each condition (lowest ΔG) is shown here. *C*, distances characterizing the YER motif (measured as the distance between Cδ of E386 to Cα of Y361 and Cδ of E386 to Cζ of R439) and their stability as measured by frequency of each distance are plotted as a 2D histogram and represented in the *side panels*. HEK293, human embryonic kidney 293 cell line; MMPBSA, Molecular Mechanics Poisson–Boltzmann Surface Area approach; OCT1, organic cation transporter 1; YER motif, Y361, E386, and R439.

In contrast, the Y361A mutant did not show uptake of pirbuterol (Fig. 1*C*)—another beta_2_-adrenergic agonist with chemical structure similar to fenoterol—at any of the concentrations tested (Fig. S2). We hypothesize that fenoterol possesses a chemical motif that rescues the transport in the Y361A mutant, which pirbuterol is lacking. In order to elucidate the motif, we used a broad spectrum of additional beta_2_ agonists with similar chemical structures and analyzed their transport by the Y361A mutant relative to the wildtype. All tested beta_2_ agonists were good OCT1 substrates (Fig. 5). OCT1 wildtype showed increased uptake ranging from 4.6-fold (salbutamol) to 77-fold (pirbuterol) compared with empty vector control cells (pcDNA5) (Fig. S3). The Y361A mutant transported dobutamine, ractopamine, and fenoterol at levels ranging from 40% to 90% compared with the wildtype (Fig. 5*A*). In contrast, the remaining beta_2_ agonists, including orciprenaline, salbutamol, and pirbuterol, were not transported (maximum 10% of wildtype activity, Fig. 5*A*). The consistent difference between the substrates that were transported and those lacking transport by the Y361A mutant was the absence of the second aromatic ring.

To gain structural insight into the ability of Y361A to transport fenoterol but not pirbuterol, we performed docking and MD simulations. The MD simulations revealed that fenoterol adopts a rotated position in the Y361A mutant. In this orientation, the resorcinol ring interacts with E386, whereas the phenol ring is positioned within the pocket created by the absence of Y361 in the Y361A mutant (Fig. 5*B*, II). Thus, the second aromatic ring of fenoterol effectively compensates for the missing Y361 in the Y361A mutant. As pirbuterol only carries one aromatic ring, which interacts with E386, it cannot in addition replace the missing aromatic ring of tyrosine in the Y361A mutant (Fig. 5*B*, IV). When summarizing all MD simulations and analyzing the distances between each amino acid of the YER motif in the wildtype and the AER motif (mutated YER motif) in the Y361A mutant, FNT showed a stable interaction within both OCT1 systems (Fig. 5, *C* and *I* and II). In contrast, pirbuterol showed a stable interaction only with the wildtype (Fig. 5*C*, III) transporter. For pirbuterol, the AER interaction motif in the Y361A mutant was disrupted in 50% of the simulations (Fig. 5*C*, IV), whereas in the presence of fenoterol, it was stable in all (100%) simulations (Fig. 5*C*, II), supporting the experimental data. It should be noted that the end poses of the MD simulations revealed that E386 is not directly interacting with the charge of fenoterol or pirbuterol, neither in the wildtype nor in the Y361A mutant (Fig. 5*B*).

**Figure 6:**
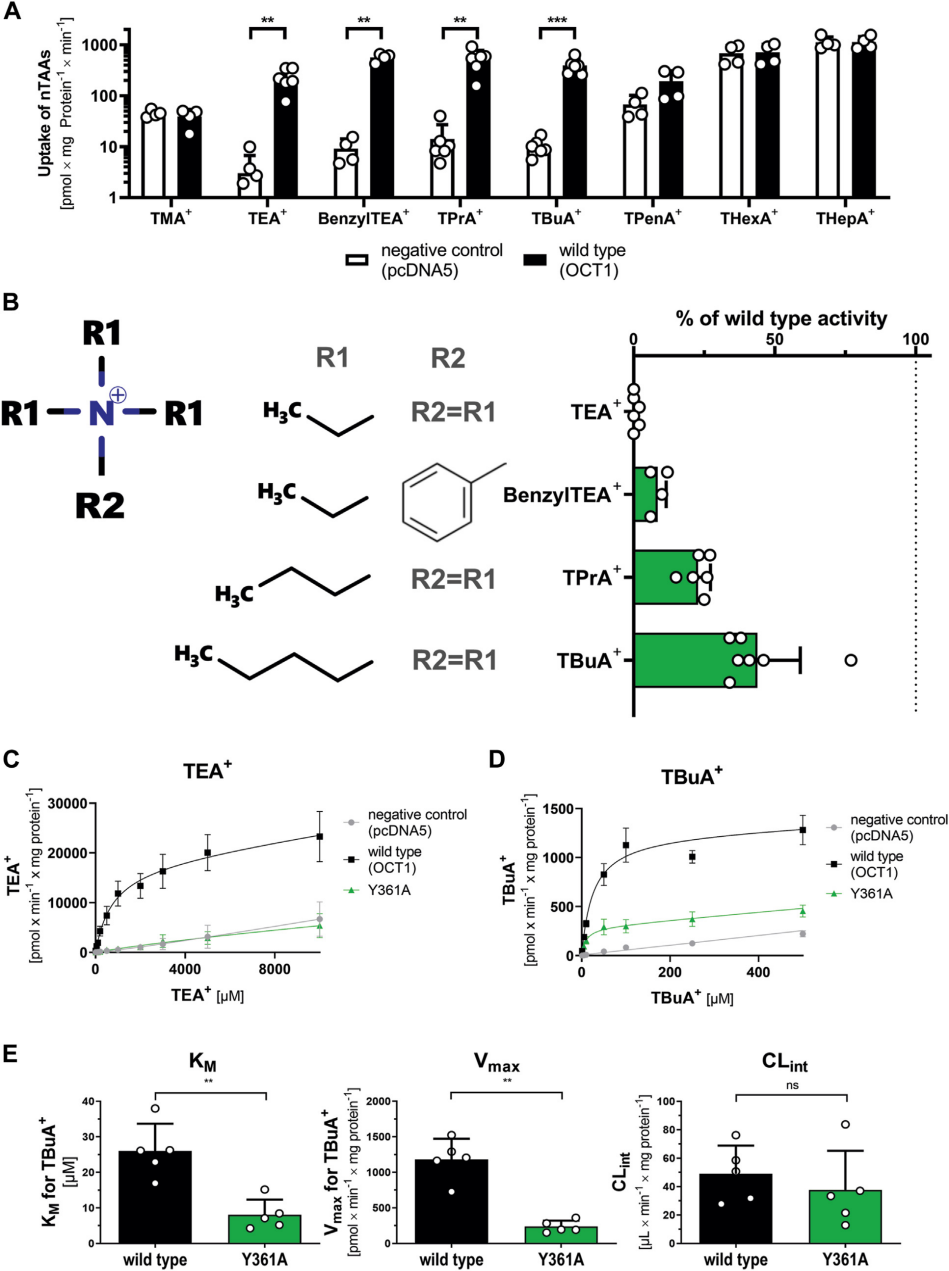
**Influence of ligand structure on the transport of tetraalkylammonium compounds (nTAAs) by the Y361A mutant**. *A*, verification of OCT1-mediated uptake of different nTAAs in HEK293 cells stably overexpressing wildtype OCT1 or empty vector control cells (pcDNA5); uptake of individual compounds was compared between wildtype and control cells using two-tailed paired *t* tests; only compounds that showed significant uptake by the wildtype (∗∗*p* < 0.01, ∗∗∗*p* < 0.001) were considered as substrates for subsequent experiments; the *y*-axis is logarithmically scaled. *B*, uptake of different nTAAs in HEK293 cells stably overexpressing the Y361A mutant; active uptake was normalized on wildtype activity after subtraction of passive diffusion into empty vector control cells (pcDNA5); concentration-dependent uptake of TEA^+^*C*, and TBuA^+^*D*, in stably transfected HEK293 cells. *E*, resulting differences in TBuA^+^ kinetics between wildtype and Y361A mutants in *K*_*M*_, *v*_max_, and CL_int_; two-tailed paired *t* tests (∗∗*p* < 0.01, ns not significant) shown are means ± SD of n = 3 to 7 independent experiments. CL_int_, intrinsic clearance; HEK293, human embryonic kidney 293 cell line; OCT1, organic cation transporter 1; TBuA^+^, tetrabutylammonium; TEA^+^, tetraethylammonium.

In addition to fenoterol, also TBuA^+^ was able to at least partially rescue the transport activity of the Y361A mutant (Fig. 1*C*). However, the related tetraalkylammonium compound (nTAA) TEA^+^ as model OCT1 substrate was not transported by Y361A. Since TBuA^+^ is a quaternary amine without any aromatic structures, we next analyzed how the length of the alkyl chain in quaternary amines affected their transport by the Y361A mutant. We examined the uptake of nTAAs with varying length of side chains ranging from methyl to heptyl and found that only compounds with a side-chain length between ethyl and butyl were transported by wildtype OCT1 (Fig. 6*A*). The transport ranged from 38-fold increase for TBuA^+^ to 52-fold increase for TEA^+^ compared with negative control cells, with transport activity decreasing as the side-chain length increased. The uptake by Y361A increased with increasing alkyl chain lengths of the substrate (Fig. 6*B*). While the smallest nTAA compound, TEA^+^, was not transported, the largest tested compound, TBuA^+^, was taken up to 38.7% by the Y361A mutant compared with the wildtype. More importantly, increasing lipophilicity with increasing alkyl chain length contributed more to the ability to be transported by the Y361A mutant than the presence of an aromatic ring, as observed with Benzyl-TEA^+^. The effects of the alkyl chain length were present over the complete concentration spectrum for TEA^+^ (Fig. 6*C*) and TBuA^+^ (Fig. 6*D*). Concentration-dependent uptake of TBuA^+^ revealed differences in the uptake kinetics between the Y361A mutant and the wildtype transporter (Fig. 6*E*) similar to the ones observed for fenoterol. *v*_max_ and *K*_*M*_ of TBuA^+^ uptake were reduced in Y361A compared with the wildtype by 4.5-fold (*p* = 0.0019) and 2.6-fold (*p* = 0.0077), respectively (Fig. 6*E*). However, this only marginally affected CL_int_ (*p* = 0.09). This suggests that large lipophilic residues in the substrate, which can but must not include an aromatic ring, may complement the gap of the missing tyrosine residue at position 361 and rescue Y361A activity.

Amisulpride was also transported by the Y361A mutant (Fig. S5), but the effects of the mutation on transport kinetics differed compared with fenoterol and TBuA⁺. The Y361A mutant transported amisulpride with 7.9-fold lower affinity and 1.9-fold higher velocity compared with the wildtype. Consequently, CL was significantly decreased by 4.1-fold (*p* = 0.008) compared with the wildtype.

**Figure 7:**
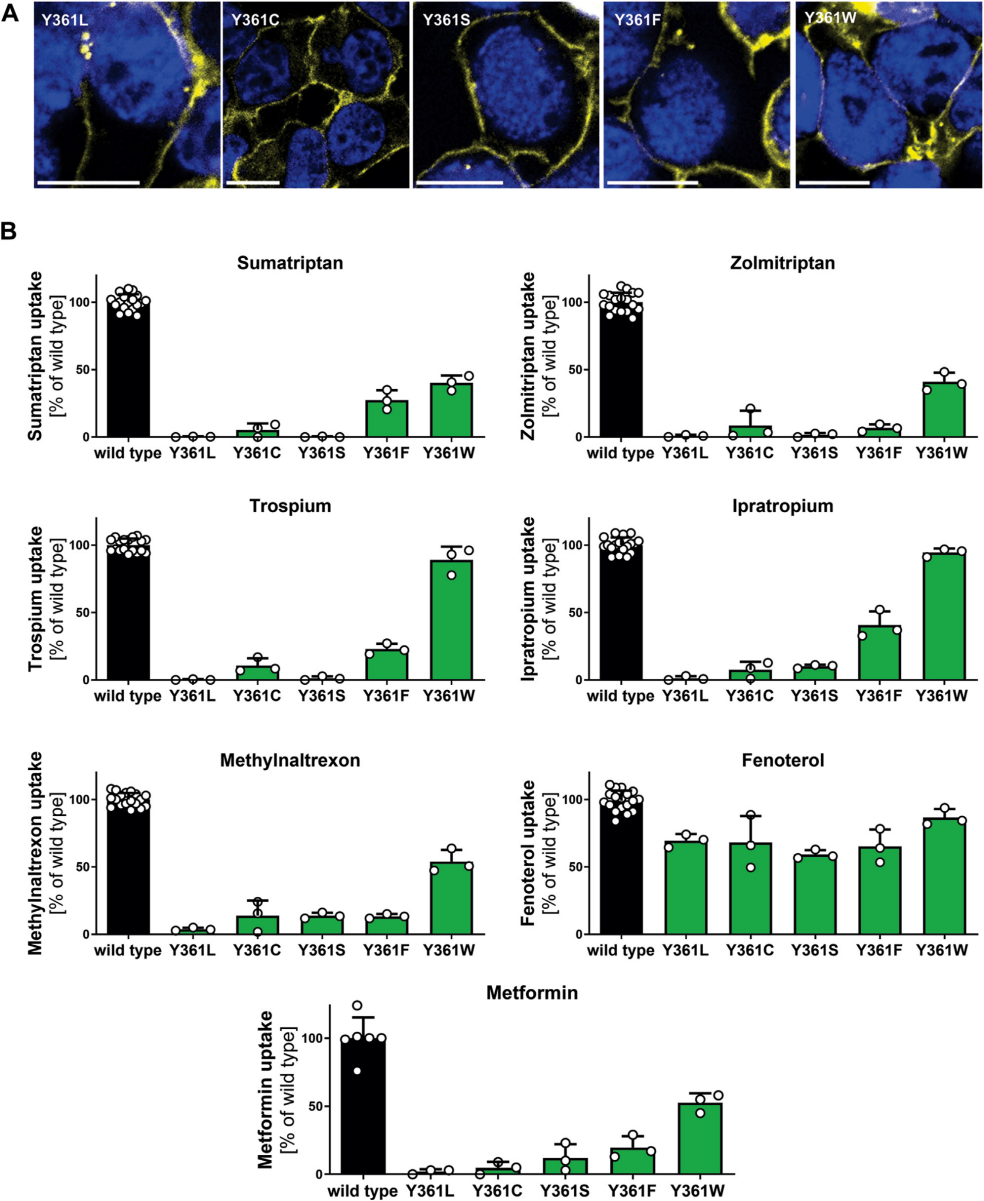
**Effects of Y361 mutation on transport of multiple OCT1 substrates**. *A*, verification of membrane localization of Y361 mutants in transiently transfected HEK293 cells; scale bar represents 10 μm. *B*, uptake of key OCT1 substrates in HEK293 cells transiently transfected with Y361 substitutions; active uptake was normalized on wildtype activity after subtraction of passive diffusion into empty vector control cells; shown are means ± SD of n = 3 independent experiments. HEK293, human embryonic kidney 293 cell line; OCT1, organic cation transporter 1.

To validate the specific role of the side chain at codon 361, we generated additional mutants to reintroduce single properties shared by tyrosine (Fig. 7). First, we mutated Y361 to leucine (Y361L) to introduce a large hydrophobic, but not aromatic, side chain. The mutant was correctly localized in the plasma membrane (Fig. 7*A*), but leucine was not able to restore transport activity for most of the tested substances (Fig. 7*B*). Second, we mutated Y361 to cysteine (Y361C) and serine (Y361S) to reintroduce a polar side chain able to participate in hydrogen bond interactions. Again, the mutants were correctly localized in the plasma membrane (Fig. 7*A*), but both were not able to restore transport activity (Fig. 7*B*). Finally, we replaced the aromatic side chain by mutating Y361 to phenylalanine (Y361F) and tryptophan (Y361W). The Y361W mutation partially or completely rescued transporter function, depending on the substrate. In contrast, Y361F only rescued between 7% (zolmitriptan) to 41% of transport activity (ipratropium, Fig. 7*B*). As expected, fenoterol uptake was not affected by any of the generated mutations. These results highlight the critical role at position 361 of both of the aromatic ring conferring lipophilic shielding and the coupled hydroxyl group that confer both hydrogen donor and acceptor properties.

### Functional interplay between Y361 (TMH7) and Y36 (THM1) in mouse OCT1

**Figure 8:**
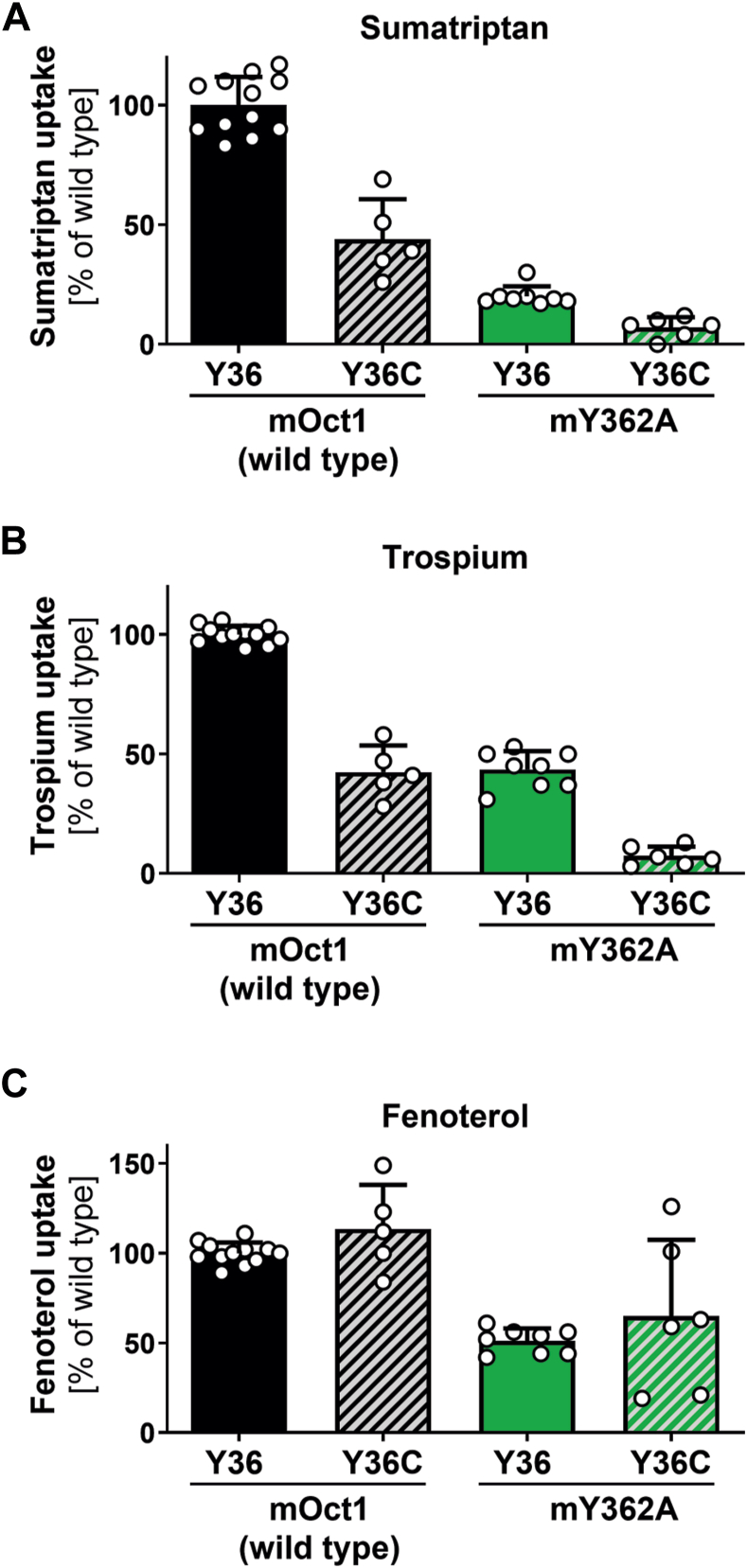
**Influence of Y36 on Y361A phenotype in mouse (murine) OCT1 (mOct1)**. Uptake of sumatriptan (*A*), trospium (*B*) and fenoterol (*C*) in HEK293 cells transiently transfected with mOct1 carrying the Y36C and Y362A substitutions alone or in combination; OCT1-mediated uptake was normalized to wildtype activity after subtraction of passive diffusion into empty vector control cells; shown are means ± SD of n = 5 to 8 independent experiments. HEK293, human embryonic kidney 293 cell line; OCT1, organic cation transporter 1.

Finally, we analyzed the involvement of the YER motif in murine Oct1 (mOct1) and a possible involvement of a thin gate between the residues at codon 361 (TMH7) and 36 (TMH1). The YER motif is highly conserved between species, including mOct1 (Fig. S6). However, one major functionally relevant difference between mouse and hOCT1 is the amino acid difference at codon 36 (20). While hOCT1 carries C36, mOct1 carries Y36. Substitution of C36Y caused strong differences in fenoterol transport kinetics (20). Suo *et al*. (21) suggested that tyrosine at position 36, which is present in mOct1, is involved in forming a thin gate with the tyrosine at position 361 (corresponding to position 362 in mOct1) because of its close proximity within the substrate-binding pocket. To analyze possible differences in the involvement of mY362 in mOct1 compared with hOCT1, we mutated Y362 to alanine in mOct1 (Y362A). Similarly to hOCT1, the mutant showed strong substrate-specific effects. Overall, the murine Y362A mutant retained an increased ability to transport substrates compared with the human mutant (Fig. 8). The mY362A mutation retained 20% to 51% of wildtype activity for sumatriptan and fenoterol, respectively. To determine whether the differences in the role of hY361–mY362 between human and mouse OCT1 can be attributed to the differences at codon 36, we substituted Y36 with cysteine (Y36C), the corresponding residue in hOCT1, to create a murine double mutant (mY36C-Y362A). This double mutation resulted in a complete loss of transport activity for all substrates tested, except for fenoterol (Fig. 8). This suggests that Y36 allows partial compensation for the missing Y362 in the mY362A mutant. Analyzing the effects of codon 36 in hOCT1, we generated a double mutant (C36Y and Y361A). Uptake was observed only for fenoterol and partially for ipratropium, indicating a more pronounced role of Y361 compared with Y36 in transport. (Fig. S7).

When mutated to alanine, the other two amino acids of the YER motif, mE387 and mR440 (corresponding to E386 and R439 in humans, respectively) showed similar effects as observed for hOCT1 (Fig. S8). The only exception is that in mouse, the mR440A mutant showed residual uptake of trospium but not of fenoterol (Fig. S8*B*).

## Discussion

In this study, we investigated the function of the YER motif, consisting of Y361, E386, and R439, in OCT1 transport. Our data show that mutation of Y361, located centrally in the binding pocket of the OCT1 transporter, exhibits substrate-specific effects. In contrast, the removal of the negative charge from E386 or the positive charge from R439 completely abolished or reduced OCT1 transport activity by more than 60%, respectively (Fig. 1).

The interaction between E386 and R439 is essential but not sufficient for OCT1 transport. Mutating E386 to the shorter aspartate, while preserving its charge, fully abolished transport (Fig. 2). This highlights the importance of the spatial position of the negatively charged carboxylic moiety of E386 relative to its Cα atom, which is most likely linked to its distance to R439 and the capability to form a salt bridge. This finding is corroborated by the R439K mutant, which preserves the charge of R439. Lysine is also by one methylene shorter than arginine, but the positive charge is centered at the amino group, whereas it is dislocated in the guanidinium group of the arginine. The electrostatic attractive force is thereby slightly larger in lysine at small distances. The cryo-EM structures showed the two amino acids in close proximity and indicated a change in relative distance in response to the transition between the inward-facing and outward-facing states (from 3.9 to 7.1 Å among all structures resolved excluding those with inhibitors) (21, 22, 23). We infer from our data and the cryo-EM structures, that both, the formation of the salt bridge for stabilizing the inward-facing state as well as the need to open the salt bridge for reaching the outward-facing state, are essential features of the mechanics of the transport cycle of OCT1. A second salt bridge formed between D474 and K214 was already shown to be essential for OCT1 function (21). This suggests that the salt bridges between D474 and K214 and between E386 and R439 act as gatekeepers, balancing the energetic stability between the inward-facing and the outward-facing states of the OCT1 substrate-binding pocket.

Interestingly, charge reversal did not restore (not even partially) transport function for any of the substrates tested (Fig. 3). This suggests additional interactions in which at least one of the two amino acids is involved. E386 could participate in interactions with the nearby located residues T245 and Q241 and also with Y361 (22, 23). Shortening the side chain could disrupt this interaction network and consequently disable transport function. A similar interaction network may also be important for R439, involving residues like not only S382 and T442 but also Y361 (22, 23).

Previously, a direct interaction between the positive charge of the substrates and the negative charge of E386 was suggested to be essential for OCT1 transport (21). This interaction might be feasible for substrates with positive charges near the ends of their molecules, like sumatriptan, ipratropium, or ranitidine. A comparable interaction is more difficult to establish for substrates like fenoterol or thiamine, where the positive charge is located in the middle of the molecule. However, our findings demonstrate the functional involvement of E386 across all tested substrates, regardless of the location of the charge (Fig. 1). Therefore, our data question the importance of a direct interaction between E386 and the substrate but instead highlight the formation of a salt bridge with R439 and an interaction network with other amino acids as essential for OCT1 function. Furthermore, our docking and MD simulation studies did not suggest any stable poses where a direct interaction between E386 and the positive charge of fenoterol was possible (Fig. 5).

Other arguments against a direct interaction of E386 with the positive charge of the substrate are first, the negative charge of E386 is conserved across orthologs and, more importantly, is present in paralogs, such as organic anion transporters (Fig. S6*A*). Even organic anion transporters that exhibit specificity for negatively charged substrates contain a negatively charged glutamate or aspartate at the corresponding position, which minimizes the potential for E386 to directly participate in substrate interactions while highlighting its essential role in the structural network maintained by the YER motif. Second, most of the cryo-EM structures resolving organic cation transporters in a substrate-bound conformation did not indicate a direct interaction of the positive charge of the substrate with E386 (22, 23, 25). Finally, and most importantly, the E386A mutation affected the uptake of noncharged substrates like zalcitabine in a similar way as it affected the uptake of charged ones (Fig. 2*C versus* 2*D*).

A similar involvement of a negatively charged residue with the substrate charge was proposed for D474 before the first OCT structure was resolved (18, 19). Similar to the effects observed for the E386A mutant, the removal of the charge of D474 led to a complete loss of uptake. The cryo-EM structures then revealed that D474 forms a salt bridge with K214 rather than directly interacting with the substrate (21), explaining the loss of function because of1 interference with the mechanics of the transporter.

Our data point to the essential role of Y361, the third residue of the YER motif. Generated Y361 mutants showed gradual and substrate-specific effects, highlighting its role in substrate recognition (Fig. 1). The Y361A mutant showed a loss of transport function for substrates with only one aromatic ring system, whereas the beta_2_-agonistic drugs possessing a second aromatic ring (Fig. 5) or nTAA compounds with four long aliphatic side chains (Fig. 6) were still transported. A hydrophobicity analysis of OCT1 showed that the aromatic ring of Y361 significantly contributes to the hydrophobicity of the binding pocket and the thin gate, which is responsible for closing the binding pocket (Fig. S9).

In the Y361A mutant, substrates can compensate for the missing tyrosine by either fitting an aromatic ring into the gap left by the mutant, as seen with fenoterol (Fig. 5), or by increasing lipophilicity within the binding pocket, as observed with TBuA^+^. Furthermore, MD simulations (Fig. 5) suggest that these substrates enhance the likelihood of triggering the closure of the upper gate of OCT1 during the transition from outward to inward states. Consequently, our data suggest that the reduced hydrophobicity by the Y361A mutant hinders closure of the outer gate, as Y361 contributes centrally to seal the substrate-binding pocket of the transporter from the extracellular environment in the occluded and inward-facing states. It should be noted that the localization of fenoterol differs between our MD simulations and the previously resolved cryo-EM structures with fenoterol (22). These differences might be attributed to the high symmetry of fenoterol, which complicates the identification of the precise positioning of its aromatic rings.

The distinct effects of the Y361A mutant on the transport of amisulpride, compared with fenoterol and TBuA⁺, suggest a different mode of interaction between amisulpride and the Y361A mutant (Fig. S5). One possible explanation may be that, compared with fenoterol and TBuA⁺, amisulpride has a higher polarity and therefore cannot functionally replace Y361 based on hydrophobicity alone. Amisulpride contains a sulfonyl group, which may instead be involved in additional polar interactions. As a result, amisulpride may bind in an unfavorable position within the substrate pocket, reducing its affinity compared with the wildtype. However, pocket closure and transport are still triggered sufficiently—or even more effectively—by these additional interactions.

To narrow down the precise mechanism by which Y361 may affect OCT1 function, we mutated Y361 to serine, cysteine, leucine, phenylalanine, and tryptophan and measured transport activity for several substrates (Fig. 7). If we focus on the substrates strongly affected by the Y361A mutation, OCT1 showed reductions of transport dependent on the Y361 mutation introduced. The leucine mutant, which introduces a bulky hydrophobic but nonaromatic amino acid, showed the strongest reduction in transport activity of all mutations. In contrast, phenylalanine and tryptophan substitutions maintained most transport activity. Phenylalanine, which differs from tyrosine only by the hydroxyl group in the para position of the aromatic ring, was inferior to the tryptophan mutant in retaining wildtype activity. From these data, we infer that OCT1 requires an aromatic ring system at position 361 and a hydrogen bonding functionality, as present in tyrosine or tryptophan but absent in leucine or phenylalanine. The most likely hydrogen bonding partner is E386, as cryo-EM structures and MD simulations showed hydrogen bonds between these two residues.

**Figure 9:**
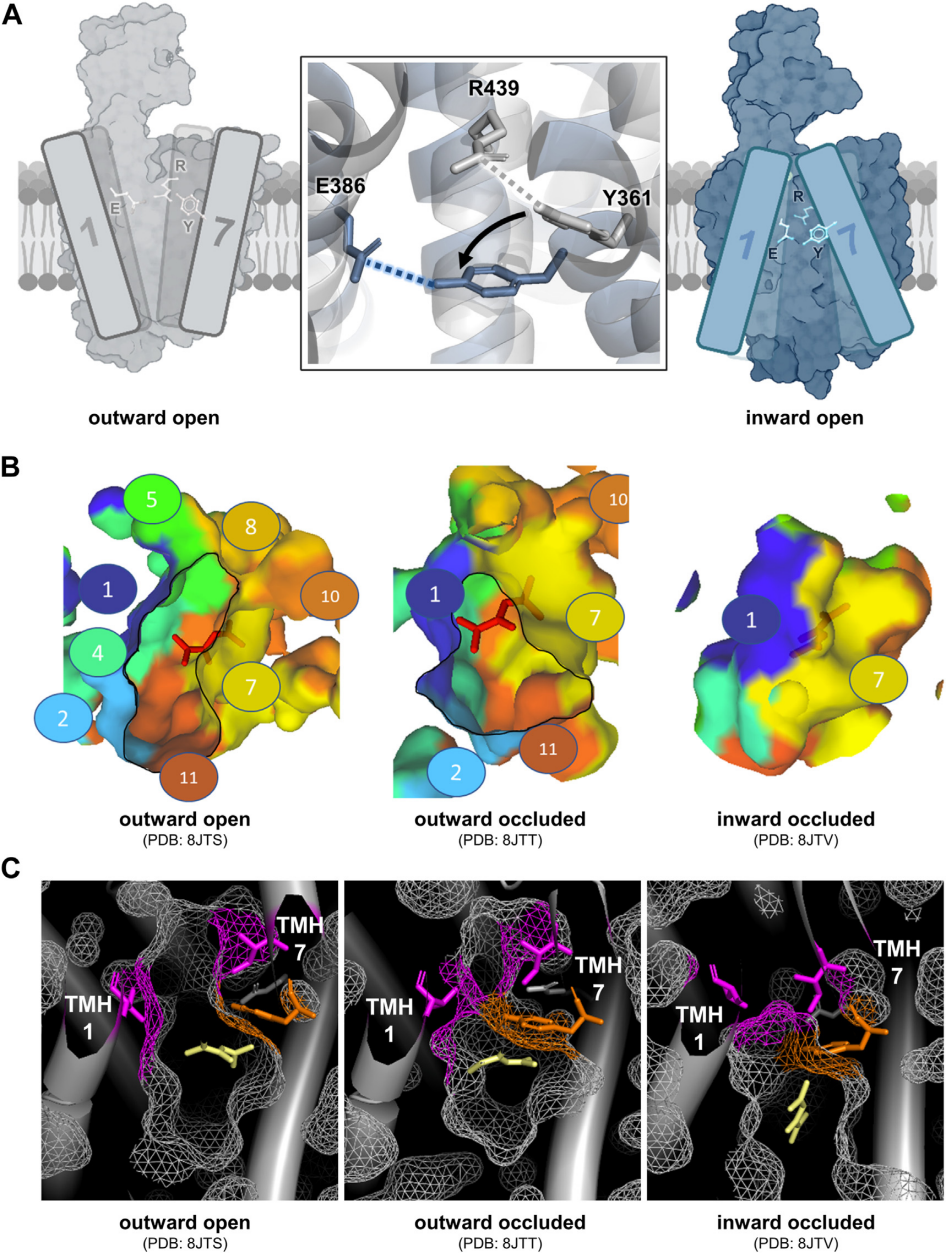
**Influence of the YER motif on closing of the substrate-binding pocket during substrate transport**. *A*, schematic representation of the switch in interaction of Y361 (Y) from R439 (R) in the outward-open state (*gray*) to E386 (E) in the inward-open state (*dark blue*), leading to the movement of TMH7 toward TMH1. *B*, surface representation of the substrate-binding pocket in different conformational states of OCT1. Different colors indicate the contribution of each transmembrane helix (TMH) to the formation and closure of the substrate-binding pocket. The view is oriented from the extracellular side. *C*, detailed resolution of amino acids involved in closing of the substrate-binding pocket (depicted as *magenta*); Y361 is represented in *orange*; metformin is represented in *pale yellow*. OCT1, organic cation transporter 1; YER motif, Y361, E386, and R439.

During the transport process, Y361 switches from interacting with R439 alone to interacting with both R439 and E386 (23). Analyses of the existing cryo-EM structures from different groups suggest that in the outward-open and outward-occluded conformation, Y361 primarily interacts with R439 (Table 1). The partial ability of the R439A mutant to transport FNT (Figs. 1 and 3) may indicate that larger lipophilic substrates with two ring systems can mimic this Y361–R439 interaction. The same cryo-EM analyses suggest that in the inward-open and inward-occluded conformations, Y361 may primarily interact with E386 (Table 1). Consistently, structures resolved in different conformations by the same group using the same OCT1 substrate show motions of Y361 and R439 toward E386 during transition from outward to inward-open conformation (Fig. 9*A*, Fig. S10). This results in the formation of short distance (thus strong) interactions of E386 with Y361, suggesting that this hydrogen bond between Y361 and E386 is a key feature of substrate transport by OCT1. These interactions can also explain why the charge reversal of E386–R439 was nonfunctional.

**Table 1:** Interaction of Y361 in different conformations demonstrating the switch in interaction from R439 toward E386

Conformation	Ligand	Interaction partner of Y361	PDB ID
Outward open	Apo	R439	8ET6
	Metformin	R439	8JTS
Outward occluded	Metformin	R439	8JTT
Inward occluded	Metformin	R439	8JTV
Inward partially open	Apo	E386	8JTW
	Apo	None	8JTY
Inward open	Apo	None	8JTX
	Apo	E386	8SC1
	Metformin	E386 + substrate	8SC4
	Fenoterol	E386 + substrate	8SC3
	Thiamine	E386	8SC6

Available cryo-EM structures resolving OCT1 (apo or substrate bound, (21, 22, 23)) were analyzed for interaction partners of the Y361 residues using the interaction analyses of the PDB database (www.rcsb.org).

Y361 may be a key lever moving TMH7 through interaction with TMH1, thereby initiating substrate transport. The cryo-EM structures showed that TMH7 and TMH1 are involved in closing the substrate-binding pocket during the transition from the outward-occluded state to the inward-occluded state (Fig. 9, *A* and *B*). Thereby, the substrate-binding pocket is closed from the extracellular space by I365 from TMH7 and by C36 and V37 from TMH1 (Fig. 9*C*, (22)). As I365 is located within TMH7 directly above Y361, Y361 may act as lipophilic lever that, upon substrate binding, induces movement of TMH7 toward TMH1, supported by I365. Furthermore, Y361 restricts the substrate-binding pocket and undergoes a strong downward movement during transition from outward-open to outward-occluded and inward-occluded conformation, acting as hydrophobic shield navigating or even pushing the substrate toward the intracellular lumen (Fig. S10). This effect is even more pronounced in mouse OCT1 (Fig. S6).

It was previously suggested for mouse Oct1 that Y362 (corresponding to human Y361) interacts directly with Y36 to close the outer gate (21). Human and mouse Oct1 differ at position 36, as hOCT1 carries a cysteine, while the ortholog transporter in mouse has a tyrosine at position 36. In our study, the effects of Y36C, Y362A, and the double mutant are consistent with both residues contributing to transport, most likely by synergistically supporting the closure of the outer gate (Fig. 8).

Interestingly, sequence analysis of the orthologs and paralogs within the organic cation transporter family shows a high conservation of an aromatic residue (tyrosine or phenylalanine) at codon 36, except for the hOCT1 and closely related primate species. Furthermore, available structures of OCT2 and OCT3 show that the corresponding residues at codon 36, tyrosine (OCT2) and phenylalanine (OCT3), contribute to sealing the substrate-binding pocket from the extracellular space. From these data, we may conclude that Y361 is directly involved in closing the substrate-binding pocket in the SLC22 transporters (Fig. S6), but as long as an aromatic residue at position 36 is present, this role is less prominent.

In conclusion, we showed substrate-dependent effects after mutating Y361 of the YER motif. This points to an important role of this amino acid in the switch from the outward state to the inward-occluded state that is essential for the transport. This also suggests that the ability of the ligand to initiate this transition may be key for the substrate specificity. However, this study does not identify a substrate-specific interaction with single amino acids in the binding pocket, suggesting a more collective property of the YER motif.

## Experimental procedures

### Reagents

The following chemicals were sourced for this study: ritodrine hydrochloride, trospium chloride, ipratropium bromide, sumatriptan-d6, serotonin-d4, and thiamine-d3 hydrochloride were obtained from Santa Cruz Biotechnology. Ranitidine-d6 and trospium-d8 were obtained from Toronto Research Chemicals. Buformin hydrochloride was supplied by Wako Chemicals. Amisulpride, metformin hydrochloride, methylnaltrexone bromide, sumatriptan succinate, frovatriptan succinate, naratriptan hydrochloride, rizatriptan benzoate, phenformin, FNT hydrobromide, dobutamine, orciprenaline hemisulfate, PRB acetate, ractopamine, salbutamol hydrochloride, terbutaline, serotonin, thiamine, trimethoprim, zolmitriptan, tetraalkylammonium compounds, norfentanyl, amisulpride-d5, and FNT-d6 were obtained from Sigma–Aldrich. All chemicals used in this study were of commercial origin and had a minimum purity of 97%.

Dulbecco’s modified Eagle’s medium (DMEM), Hanks’ buffered salt solution (HBSS), fetal bovine serum, and the Pierce Bicinchoninic Acid Protein Assay were obtained from Thermo Fisher Scientific. Penicillin–streptomycin as sourced from PAN-Biotech. Poly-d-lysine hydrobromide was purchased from Sigma–Aldrich. Hepes was supplied by Carl Roth. Acetonitrile, methanol, and formic acid, all of LC–MS/MS grade, were obtained from Merck. Twelve- and 24-well plates were obtained from Starlab, and tissue culture flasks were obtained from Sarstedt.

### Cell lines and cell culturing

All cell lines used in this work were either T-REx-293 cells (Life Technologies) or therefrom originating stably overexpressing cell lines that were generated by targeted chromosomal integration using the Flp-In System (Life Technologies) as described previously (26, 27, 28). T-Rex-293 cells do not exhibit a relevant background of endogenous OCT1 expression (27, 29, 30). Cells were cultured in DMEM supplemented with 10% fetal bovine serum, 100 U/ml penicillin, and 100 μg/ml streptomycin, maintained at 37°C with 5% CO_2_, and passaged twice weekly.

### Generation of OCT1 expression constructs

Expression constructs encoding for hOCT1 or murine Oct1 (referred to as wildtype) were fused with a C-terminal tag encoding for mVenus fluorescent protein. This allowed the direct verification of membrane localization in transfected cell lines. The vector encoding for the C-terminal tag only was synthesized by Genewiz and fused to an OCT1-encoding plasmid using the overlap extension method (31) and using primers listed in Table S1. The resulting amplicon was cloned into the pcDNA5/FRT expression vector (Thermo Fisher Scientific). The fusion protein of OCT1 with C-terminal tag was characterized to influence neither OCT1 function nor the mRNA expression (Fig. S10). These wildtype expression constructs were used to introduce point mutations by site-directed mutagenesis and mutagenesis primers as listed in Table S2. The integrity of the OCT1 expression constructs and verification of mutation sites were confirmed by capillary sequencing of the OCT1 open reading frame.

### Transient and stable transfection of T-REx-293 cells for cellular uptake experiments

For transient transfection, 5 × 10^5^ T-REx-293 cells were seeded per well in 12-well plates precoated with poly-d-lysine. Twenty-four hours later, cells were transfected. Therefore, cells were washed twice with DMEM containing 10% fetal calf serum. Then, 2 μg plasmid diluted in pure DMEM supplemented with 3% Lipofectamine 2000 (Thermo Fisher Scientific) was added per well. After 6 h, the medium was changed to full culture medium. At 48 h after transfection, efficacy was assessed by fluorescence microscopy, and cellular uptake experiments were conducted subsequently. All generated mutants were initially characterized in transiently transfected cells. To enable more in-depth analysis of the effects of the YER motif on transport, cell lines stably overexpressing the OCT1 alanine mutants (E386A, R439A, and Y361A) were generated by targeted chromosomal integration using the Flp-In System as described before (26, 27, 28).

### Cellular uptake experiments in cell lines stably overexpressing OCT1 mutants

Forty-eight hours before uptake experiments, 3 × 10^5^/6 × 10^5^ cells were seeded per well in 24-well/12-well plates precoated with poly-d-lysine. Cellular uptake experiments were conducted at 37 °C and pH 7.4 using HBSS supplemented with 10 mM Hepes, hereafter referred to as HBSS+. Substrates and concentrations used are listed in Table S3. The cells were rinsed with 0.5 ml/1 ml of prewarmed (37°C) HBSS+, and the uptake was initiated by adding 180 μl/500 μl of prewarmed HBSS+ containing the substrate. After exactly 2 min, cellular uptake was terminated by adding 400 μl/2 ml of ice-cold HBSS+. Subsequently, cells were washed twice with 1 ml/2 ml of ice-cold HBSS+ and lysed using either 80% acetonitrile with an internal standard or radioimmunoprecipitation assay buffer, depending on whether samples were used for LC–MS/MS detection or determination of total protein content, respectively. Intracellular substrate concentrations were subsequently measured and normalized to the total protein content in the samples, determined by the bicinchoninic acid assay (32).

### Quantification of intracellular substrate concentration by LC–MS/MS

For LC–MS/MS quantification of intracellular substrate concentrations, the cell lysate was centrifuged at 16,000*g* for 15 min. Then, 200 μl of the supernatant was evaporated to dryness under nitrogen flow at 40 °C. The dried sample was reconstituted with 100 μl of 0.1% formic acid and further diluted in the same solvent before 3 to 5 μl was injected into the LC–MS/MS system.

LC–MS/MS analysis was performed using an API6500 QTRAP tandem mass spectrometer with an ESI interface (AB SCIEX), coupled to a Shimadzu LC-40 UHPLC system (Shimadzu). Chromatographic separation was performed on a Brownlee SPP RP-Amide column (4.6 × 100 mm, 2.7 μm; PerkinElmer) using a mobile phase of 0.1% (v/v) formic acid (solvent A) and varying concentrations of organic solvent composed of 90% methanol/acetonitrile (6 + 1) and 0.1% formic acid (solvent B, Table S4).

### Confocal microscopy analysis of OCT1-overexpressing cells

To verify membrane expression of the generated OCT1 mutants, 5 × 10^5^ T-REx-293 cells were seeded on precoated cover slips in 12-well plates and transiently transfected as described previously. Forty-eight hours after transient transfection, cells were washed 3 times with 1 ml Dulbecco’s PBS for 5 min and were then fixed with 100% ethanol for 20 min. After washing 3 times with 1 ml Dulbecco’s PBS for 5 min, cover slips were fixed onto microscope slides using ROTI mount FluorCare DAPI (Carl Roth). Cells were analyzed using the laser scanning microscope LSM780 (Carl Zeiss) with ZEN 2010 software, version 6.0. The microscopy images were captured using a 64x objective, and individual areas of the microscopy field were enlarged using the crop function. Images were adjusted for contrast and brightness using the Fiji distribution of ImageJ2 (33, 34).

### Ligand parameterization

The 3D structures of fenoterol and pirbuterol were constructed using the YASARA build function based on their respective SMILES strings, which included stereochemical information (35). Ligands were protonated to pH 7.4 and parameterized using a custom workflow that employed Open Babel (36), Obminimize, and ACPYPE (37). The GAFF2 force field (38) was used for energy minimization, and GROMACS-compatible topology files were generated with included position restraints for MD simulations.

### Molecular docking

Docking simulations were performed using the inward-occluded conformation of OCT1 modeled from the cryo-EM structure (Protein Data Bank [PDB] ID:8JTV). The binding pose of verapamil from the OCT1–verapamil cryo-EM structure (PDB ID: 8ET8) was used to guide the definition of docking constraints. Ligands FNT and PRB were docked into the binding pocket using GOLD (39) (The Cambridge Crystallographic Data Centre) with the ChemPLP scoring function. Docking constraints included a similarity constraint based on verapamil’s pose and a pharmacophore constraint defining residue Y361 as participating in a hydrogen bond. The resulting poses were evaluated for plausibility and used as starting configurations for MD simulations.

### MD simulations

The inward-occluded conformation of OCT1 (PDB ID: 8JTV) was embedded in a lipid bilayer composed of 45% 1-palmitoyl-2-oleoyl-*sn*-glycerol-3-phosphocholine, 10% 1-palmitoyl-2-oleoyl-*sn*-glycero-3-phosphoethanolamine, 5% 1-palmitoyl-2-oleoyl-*sn*-glycero-3-phospho-l-serine, and 40% cholesterol, solvated with TIP3P water and 150 mM NaCl. A coarse-grained representation was generated using the MARTINI force field (40, 41, 42) and equilibrated over 10 μs with a time step of 20 fs while restraining the protein.

The system was converted to an all-atom representation using the MARTINI-to-atomistic conversion protocol (43), replacing the transporter with the original OCT1 structure to avoid inaccuracies. Overlaps between the protein and membrane were resolved using the Membed procedure (44). The Amber ff99SB-ILDN force field (45) was applied to the protein, ions, and solvent, whereas Slipid (46, 47) described the lipid components.

Energy minimization was followed by six-stage equilibration with gradually reduced position restraints (1000–1 kJ/mol/nm). Production simulations were performed in triplicate for 100 ns using GROMACS 2021.4 (48). The temperature was maintained at 310 K using a velocity-rescaling thermostat (49) and pressure at 1 bar with a Parrinello–Rahman barostat (50). Electrostatics were computed with the particle mesh Ewald method (51), and van der Waals interactions were modeled with a 0.9 nm cutoff. Simulations were performed at pH 7, which corresponds to physiological pH and ensures that both FNT and PRB were positively charged.

Simulations were analyzed to evaluate ligand stability, protein–ligand interactions, and overall protein dynamics. Key analyses included calculating ligand-protein distances using GROMACS (48). Visualization of structural data was performed using PyMOL (The PyMOL Molecular Graphics System, version 2.5.7; Schrödinger, LLC) and ChimeraX (52) whereas plots and further analyses were generated with custom Python scripts.

### Data analyses

Percentual uptake of mutants compared with the wildtype was calculated as uptake (mutant)/uptake (wildtype) after subtraction of passive diffusion obtained from empty vector controls. Kinetic transport parameters, *K*_*M*_ and *v*_max_, were calculated through nonlinear regression using the Michaelis–Menten equation in GraphPad Prism, version 8.01 (GraphPad Software, Inc). CL_int_ was calculated as *v*_max_/*K*_*M*_. Kinetic parameters were compared using two-tailed, paired *t* tests. All uptake studies of substrates at single concentration were performed in duplicates, and means were represented in graphs. In the cases where the data are represented as a percentage of the wildtype, the variation of the wildtype samples represents the variation of the two biological duplicates performed in each experiment. We used the PyMOL for visualization and determination of distances between residues of already published cryo-EM structures. Schematic visualizations were created using biorender.com (https://biorender.com/i31s738, https://BioRender.com/g23u187). Marvin was used for drawing, displaying, and characterizing chemical structures, Marvin 17.21.0, Chemaxon (https://www.chemaxon.com).

## Data availability

All data described are contained within this article and supporting information.

## Supporting information

This article contains supporting information (10, 14, 20, 21, 23, 29, 53, 54, 55, 56).

## Conflict of interest

The authors declare that they have no conflicts of interest with the contents of this article.
