# Supplementary material for: Substrate-specific effects point to the important role of Y361 as part of the YER motif in closing the binding pocket of OCT1
Source: J Biol Chem. 2025 Feb 14;301(4):108318. doi: 10.1016/j.jbc.2025.108318 (PMC12005293; doi:10.1016/j.jbc.2025.108318)
Supplement: Supplementary Materials [file mmc1.pdf]

# Supporting information

## Substrate-specific effects point to the role of Y361 as part of the YER motif in closing the binding pocket of OCT1

Sarah Römer[1], Erika Lazzarin[2], Anna Neumann[1], Erik Lindemann[1], Marleen J. Meyer-Tönnies[1], Thomas Stockner[2], Mladen V. Tzvetkov[1]*

[1] Department of General Pharmacology, Institute of Pharmacology, Center of Drug Absorption and Transport (C_DAT), University Medicine Greifswald, Greifswald, Germany

[2] Institute of Pharmacology, Center for Physiology and Pharmacology, Vienna, Austria

**Table of contents**

**Figure S1** ........ S-2
**Figure S2** ........ S-2
**Figure S3** ........ S-3
**Figure S4** ........ S-4
**Figure S5** ........ S-5
**Figure S6** ........ S-6
**Figure S7** ........ S-7
**Figure S8** ........ S-8
**Figure S9** ........ S-8
**Figure S10** ........ S-9
**Figure S11** ........ S-9

**Table S1** ........ S-10
**Table S2** ........ S-10
**Table S3** ........ S-11
**Table S4** ........ S-12

**References** ........ S-13

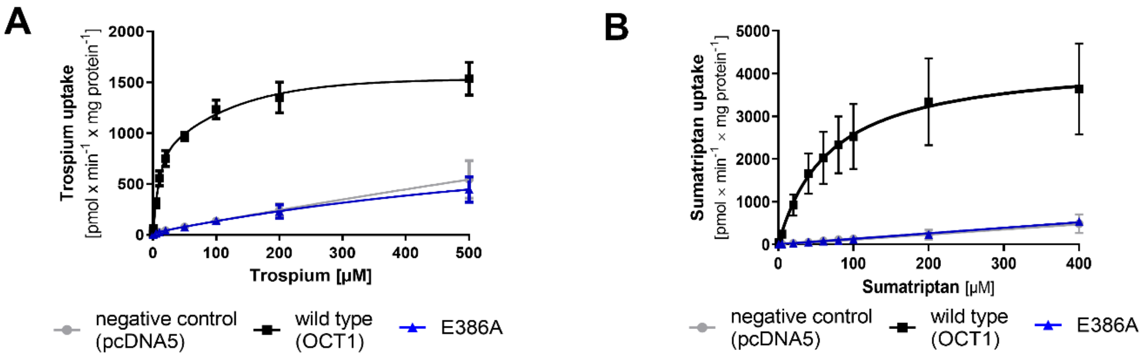


***Figure S1. Concentration-dependent uptake of trospium (A) and sumatriptan (B) by the E386A mutant*** *HEK293 cells stably overexpressing the E386A mutant were incubated with increasing concentrations of trospium (A) or sumatriptan (B) and resulting cellular substrate concentrations were measured; shown are means ±SEM of n=3 independent experiments*

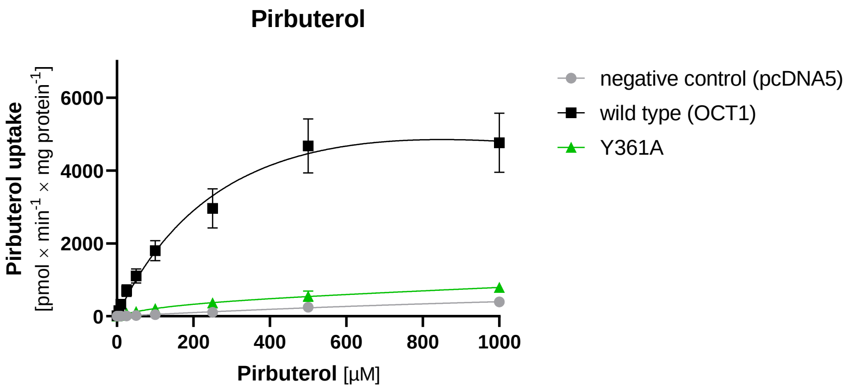


***Figure S2. Concentration-dependent uptake of pirbuterol by the Y361A mutant*** *HEK293 cells stably overexpressing the Y3616A mutant were incubated with increasing concentrations of pirbuterol and resulting cellular substrate concentrations were measured; shown are means ± SD of n=5 independent experiments*

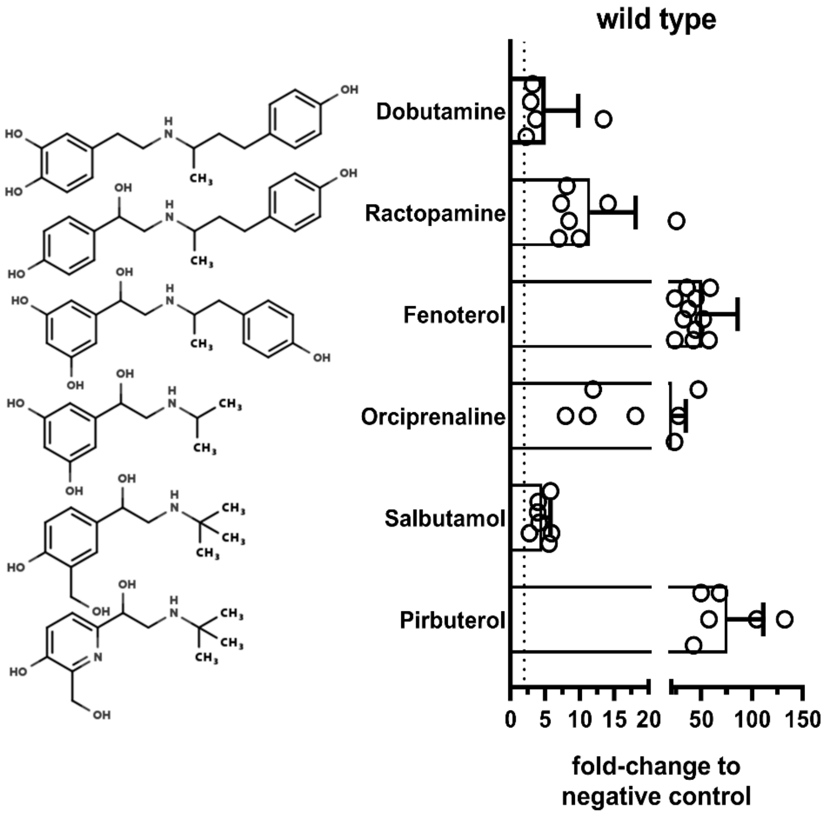


***Figure S3.*** **Uptake of β$_2$-adrenergic compounds by wild type OCT1** *Uptake of different beta2 agonists in HEK293 cells stably overexpressing wild type OCT1; active uptake is represented as fold-change to empty vector control cells; dashed line indicates 2-fold increased uptake compared to empty vector control cells as cut-off between passive diffusion (<2) and transporter-facilitated uptake (>2); shown are means, single values, and SD*

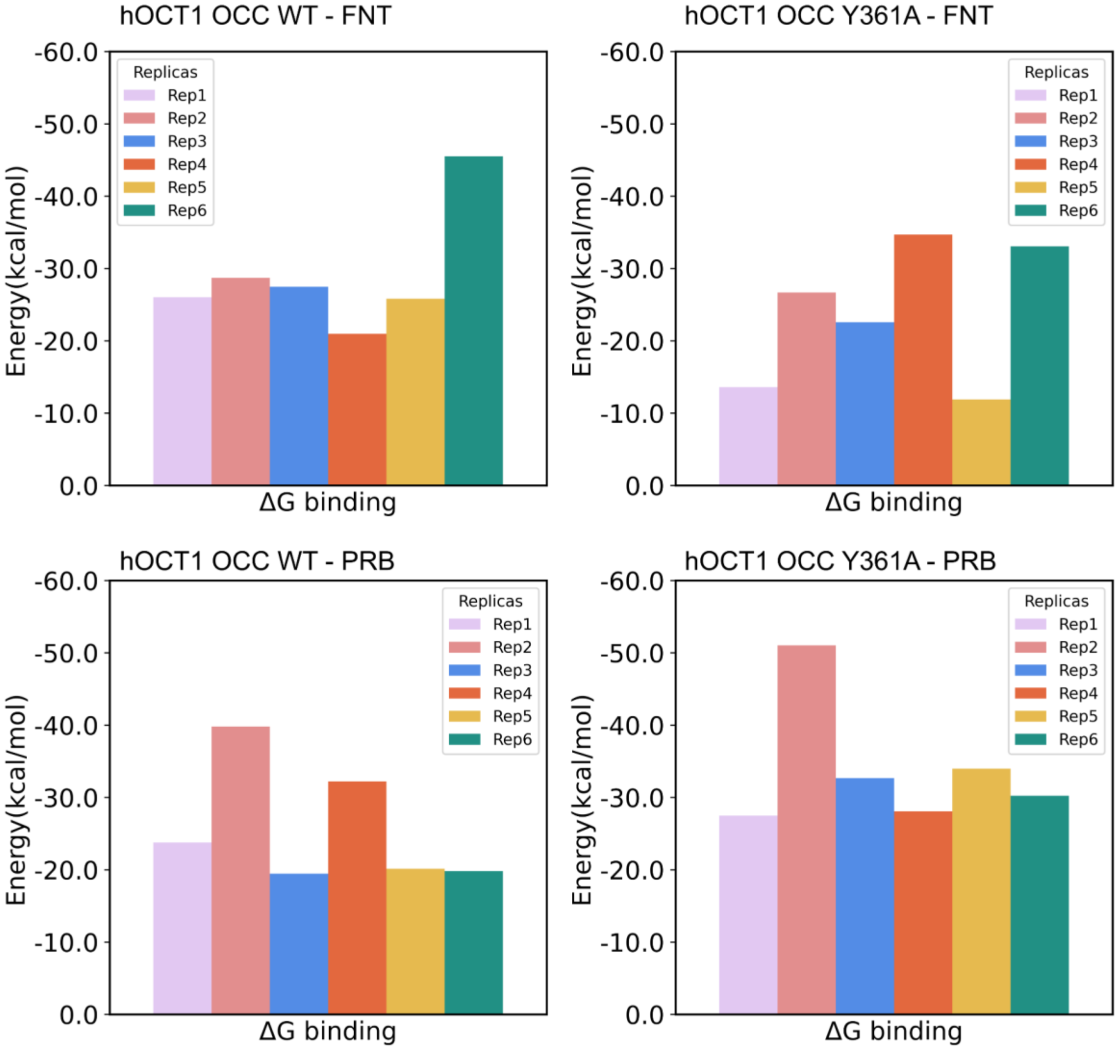


***Figure S4. Free-energy calculation of Molecular Mechanics Poison-Boltzmann Surface Area (MM-PBSA) binding energy*** *Displayed are variations in binding energies of six independent replicates; WT, wild type; FNT, fenoterol; PRB, pirbuterol*

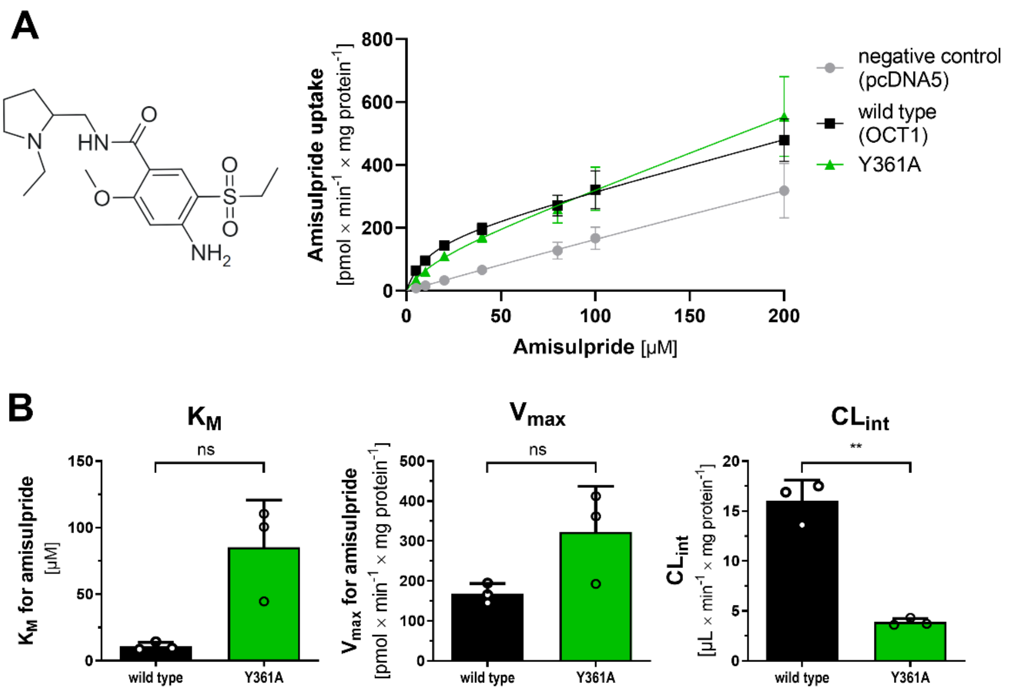


***Figure S5.*** **Effects of Y361A mutation on uptake of amisulpride** *A) Concentration dependent uptake of amisulpride in HEK293 cells stably overexpressing the Y361A mutant or wild type OCT1; empty vector control cells (pcDNA5) were used as reference for passive diffusion B) Resulting pharmacokinetic parameters; two-tailed paired t-test (** p<0.02, ns not significant); shown are means ± SD of n=3 independent experiments*

**A**

| | TMH1 36 | TMH7 361 | TMH8 386 | TMH10 439 |
|---|---|---|---|---|
| hOCT1 | …FAPICVGIV… | …DSVLYQGLI… | …SALVEIPGA… | …MCVGRMGIT… |
| mOct1 | …LAPIYVGIV… | …CAVLYQGLI… | …SSLVEFPAA… | …ACLGRMGAT… |
| hOCT2 | …FAPIYVGIV | …SSVLYQGLI… | …SALVEFPAA… | …SCLGRMGIT… |
| hOCT3 | …FAFLFVGVV | …SAVVYQGLV… | …SGVVELPGA… | …ATLGRLGIT… |
| hOAT1 | …MASHNTLQN… | …TSFAYYGLV… | …FGAVDLPAK… | …AVLGKGCLA… |
| hOAT2 | …LPLHFLLPI… | …VNFSYYGLS… | …FGAVELPSK… | …AVMGKAFSE… |
| hOAT3 | …MANHNLLQI… | …TGFAYYSLA… | …FGGVDVPAK… | …AVFGKGCLS… |

**B**

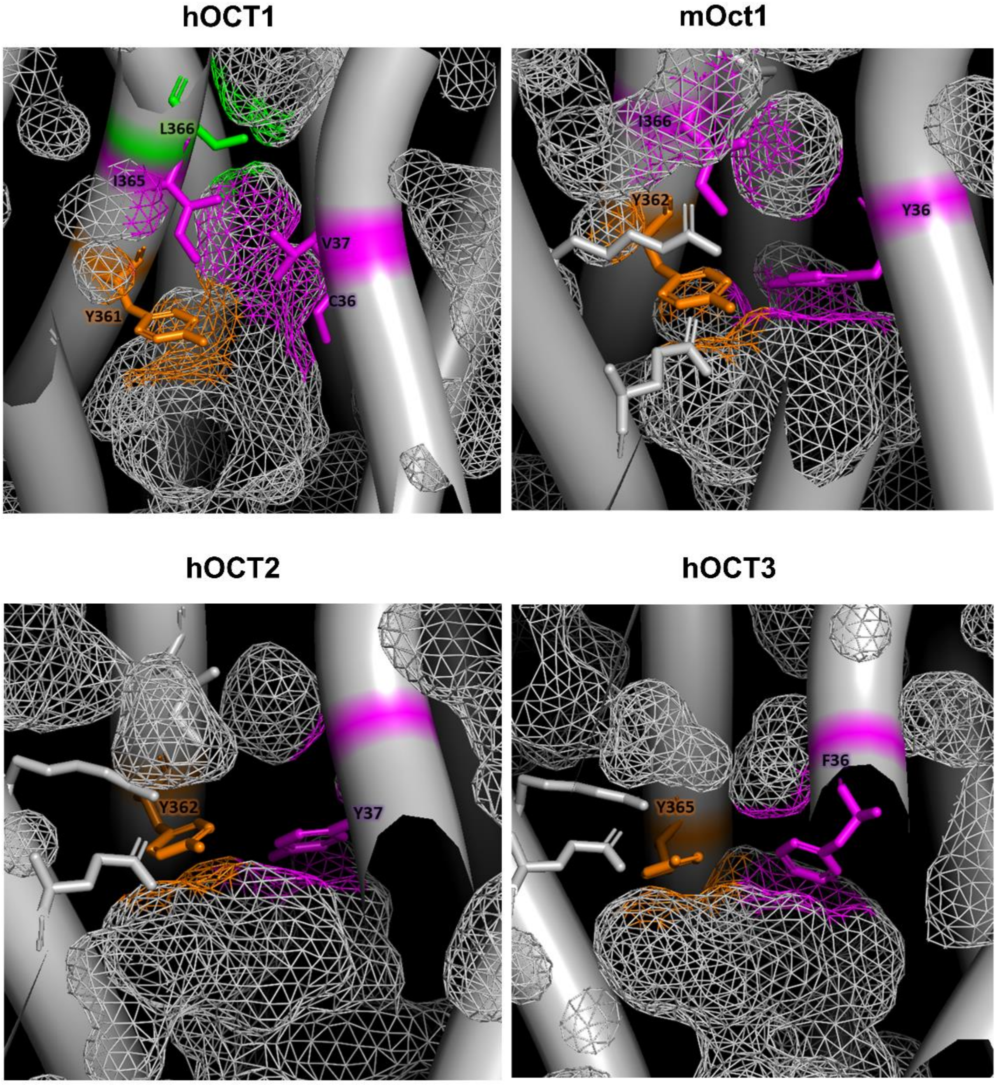


***Figure S6. Role of Y361 across orthologs and paralogs*** *A) Conservation of the YER motif across orthologs and paralogs B) Role of codon 36 as possible interaction partner with Y361 to close the substrate binding pocket; structures were generated by alpha-fold 3 (alphafoldserver.com (53)) modelling OCTs in inward open conformation; Y361 and corresponding residues are shown in orange; residues that are additionally involved in closing of the substrate binding pocket are shown in violet; E386 and R439 are shown in grey*

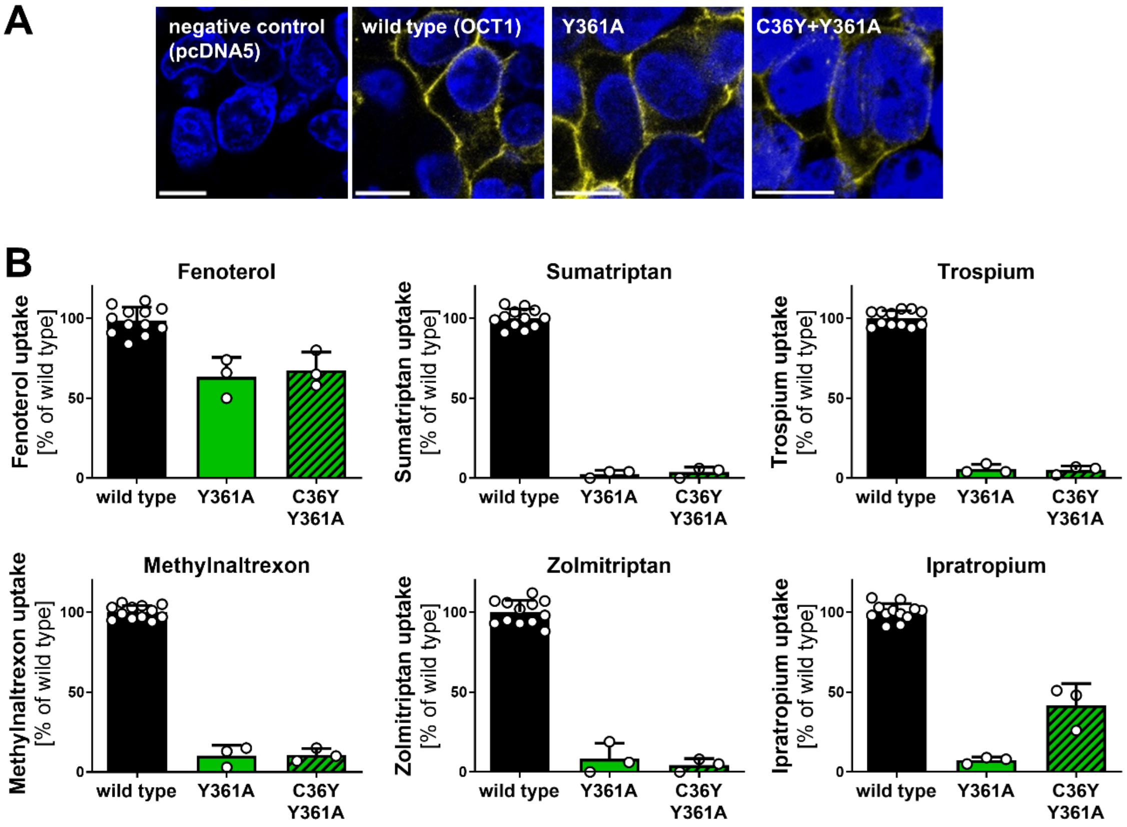


***Figure S7.*** **Influence of codon 36 on Y361A phenotype** A*) Verification of membrane localization of the C36Y (as present in mOct1) and Y361A mutants in transiently transfected HEK293 cells; scale bar 10 µm* B) *Uptake of key OCT1 substrates in HEK293 cells transiently transfected with hOCT1 carrying the C36Y and Y361A substitution; OCT1-mediated uptake was normalized on wild type activity after subtraction of passive diffusion into empty vector control cells; shown are means ± SD of n=3 independent experiments*

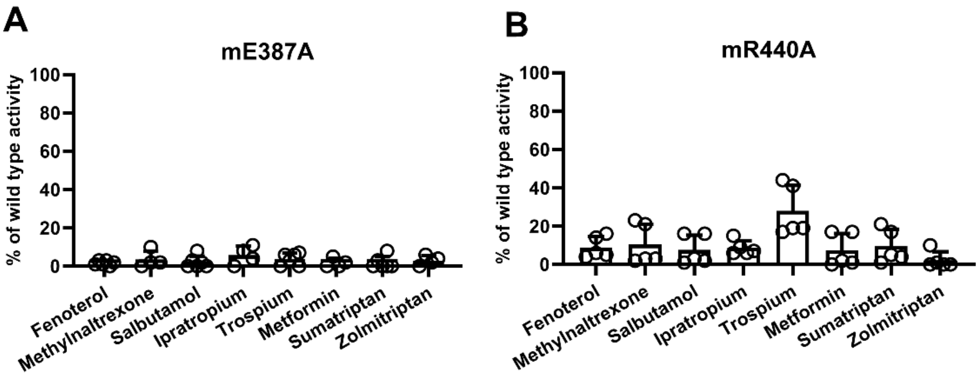


***Figure S8. Influence of E387A and R440A mutation in mOct1 on substrate uptake*** *Uptake of key OCT1 substrates in HEK293 cells transiently transfected with mOct1 carrying the E387A mutation (A, corresponding to E386A in hOCT1) or the R440A mutation (B, corresponding to R439A in hOCT1); OCT1-mediated uptake was normalized on wild type activity after subtraction of passive diffusion into empty vector control cells; shown are means ± SD of n=4-5 independent experiments*

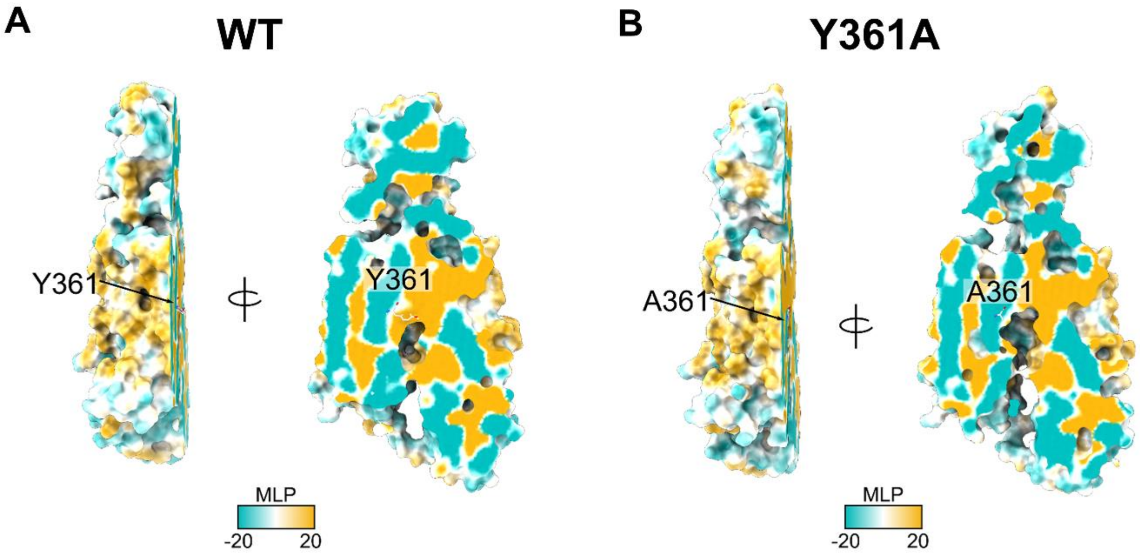


***Figure S9. Structural representation of hOCT1 in the inward-occluded state (OCC), comparing the wild type (WT) and mutant (Y361A) forms (PDB ID: 8JTV (23))*** *The protein is shown as a surface model, colored based on molecular lipophilicity (MLP) according to the provided legend*

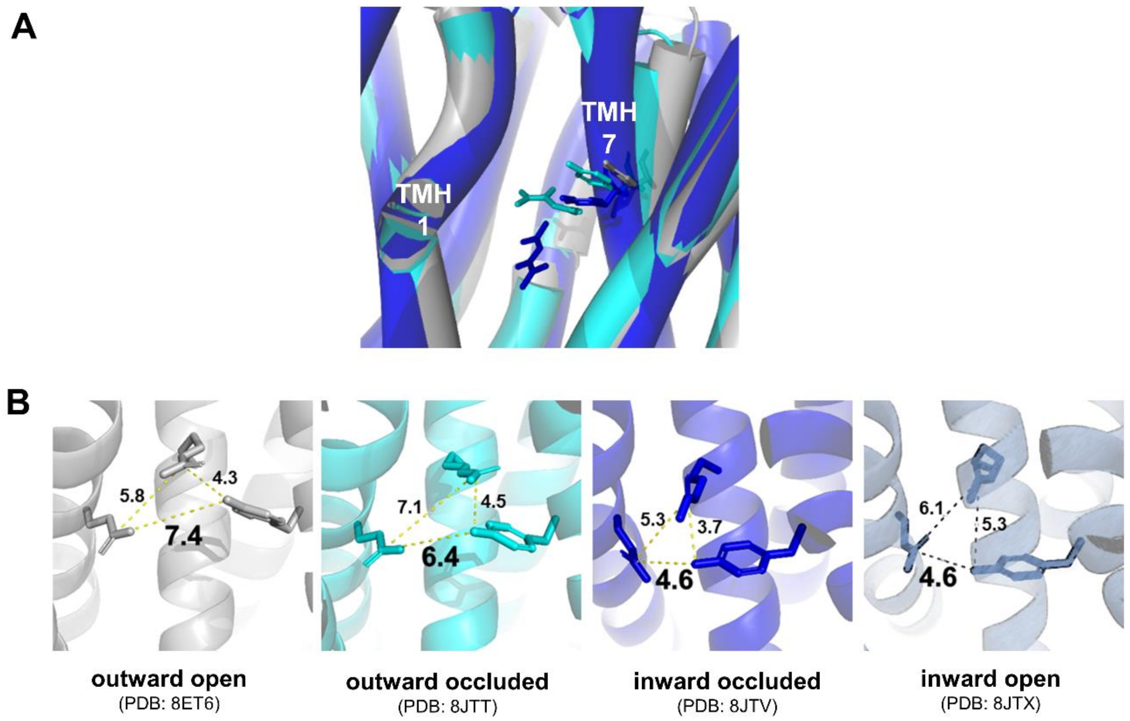


***Figure S10. Movement of YER residues during substrate-induced conformational changes of OCT1*** *A) Movement of Y361 from outward open (grey) to outward occluded (cyan) to inward occluded (blue) and associated movement of metformin B) Resulting changes in interactions between the YER residues; 8ET6 (21) was used as reference for outward open conformation as 8JTS did not resolve E386 (23); distances between residues are given in Å*

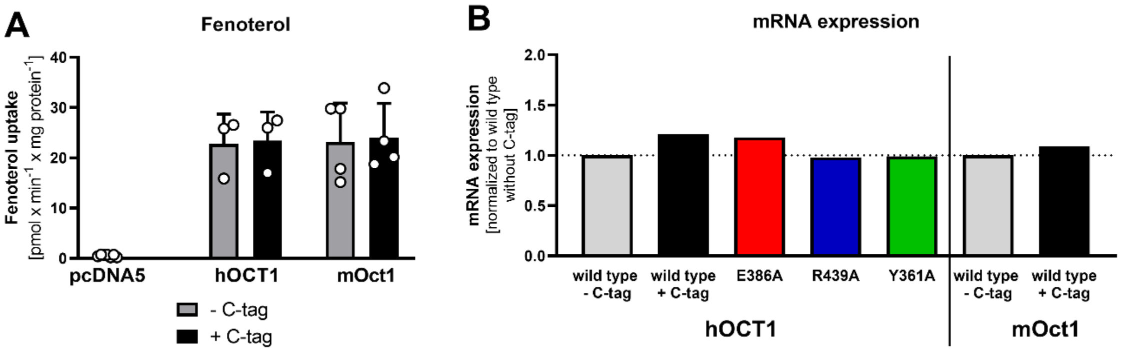


***Figure S11. Comparison of effects of c-terminal tagged fluorescence protein on OCT1 function (A) and expression (B)***

*Table S1. Primers used for generation of OCT1 expression construcs with C-terminal fluorescent tag*

| Primer name | Sequence |
|---|---|
| **pcDNA5_for** | GCGTGTACGGTGGGAGGTCTATATA |
| **C-terminus_for (hOCT1)** | TCCAAACCTCAGAACCCTCGGGCACCGGC |
| **C-terminus_rev (hOCT1)** | GGTGCCCGAGGGTTCTGAGGTTTGGACCTT |
| **C_mOCT1_for** | CAGGCAAATCCCCACATACCGGCGGAAGCGGTCTGAACGATAT |
| **C_mOCT1_rev** | TCGTTCAGACCGCTTCCGCCGGTATGTGGGGATTTGCCTGTTT |
| **pUC_rev** | GTGAGCGGATAACAATTTCACACAG |

*Table S2. Primers used for site-directed mutagenesis to introduce point mutations*

| Construct | Primer name | Sequence (5'->3') |
|---|---|---|
| **E386A** | E386A_for | CTTTACTCCGCTCTGGTCGCAATCCCGGGGGCCTTCATA |
| | E386A_rev | TATGAAGGCCCCCGGGATTGCGACCAGAGCGGAGTAAAG |
| **E386Q** | E386Q_for | CTTTACTCCGCTCTGGTCCAGATCCCGGGGGCCTTCATA |
| | E386Q_rev | TATGAAGGCCCCCGGGATCTGGACCAGAGCGGAGTAAAG |
| **E386D** | E386D_for | CTTTACTCCGCTCTGGTCGACATCCCGGGGGCCTTCATA |
| | E386D_rev | TATGAAGGCCCCCGGGATGTCGACCAGAGCGGAGTAAAG |
| **E386R** | E386R_for | CTTTACTCCGCTCTGGTCCGAATCCCGGGGGCCTTCATA |
| | E386R_rev | TATGAAGGCCCCCGGGATTCGGACCAGAGCGGAGTAAAG |
| **R439A** | R439A_for | CATAATCATGTGTGTTGGCGCAATGGGAATCACCATTGCAATAC |
| | R439A_rev | GTATTGCAATGGTGATTCCCATTGCGCCAACACACATGATTATG |
| **R439K** | R439K_for | ATAATCATGTGTGTTGGCAAAATGGGAATCACCATTGCA |
| | R439K_rev | TGCAATGGTGATTCCCATTTTGCCAACACACATGATTAT |
| **R439E** | R439E_for | ATAATCATGTGTGTTGGCGAAATGGGAATCACCATTGCA |
| | R439E_rev | TGCAATGGTGATTCCCATTTCGCCAACACACATGATTAT |
| **Y361A** | Y361A_for | TTCACGGACTCTGTGCTCGCCCAGGGGCTCATCCTGCA |
| | Y361A_rev | TGCAGGATGAGCCCCTGGGCGAGCACAGAGTCCGTGAA |
| **Y361L** | Y361L_for | TTCACGGACTCTGTGCTCTTACAGGGGCTCATCCTGCA |
| | Y361L_rev | TGCAGGATGAGCCCCTGTAAGAGCACAGAGTCCGTGAA |
| **Y361C** | Y361C_for | TTCACGGACTCTGTGCTCTGTCAGGGGCTCATCCTGCA |
| | Y361C_rev | TGCAGGATGAGCCCCTGACAGAGCACAGAGTCCGTGAA |
| **Y361S** | Y361S_for | TTCACGGACTCTGTGCTCAGTCAGGGGCTCATCCTGCA |
| | Y361S_rev | TGCAGGATGAGCCCCTGACTGAGCACAGAGTCCGTGAA |
| **Y361F** | Y361F_for | TTCACGGACTCTGTGCTCTTTCAGGGGCTCATCCTGCA |
| | Y361F_rev | TGCAGGATGAGCCCCTGAAAGAGCACAGAGTCCGTGAA |
| **Y361W** | Y361W_for | TTCACGGACTCTGTGCTCTGGCAGGGGCTCATCCTGCA |
| | Y361W_rev | TGCAGGATGAGCCCCTGCCAGAGCACAGAGTCCGTGAA |
| **mY362A** | mY362A_for | TTCTCTTGTGCTGTGCTGGCCCAGGGCCTCATCATGCAT |
| | mY362A_rev | ATGCATGATGAGGCCCTGGGCCAGCACAGCACAAGAGAA |
| **mY36C** | mY36C_for | GCTTCTTTAGCTCCCATCTGTGTGGGCATC |
| | mY36C_rev | CAGGAAAAAAACGATGCCCAGACAGATGGGAGC |

***Table S3. Substrates and concentrations used for uptake experiments*** *Concentrations were chosen to be below known $K_M$.*

| Substrate class | Substrates | Concentration used for uptake experiment [µM] | $K_M$ | Reference for $K_M$ |
|---|---|---|---|---|
| Tetraalcyl-ammonium compounds (nTAA) | Tetramethylammonium ($TMA^+$) | 10 | N/A | N/A |
| | Tetraethylammonium ($TEA^+$) | 10 | 749 | (20) |
| | Benzyl-triethylammonium (Benzyl-$TEA^+$) | 10 | 38.6 | (54) |
| | Tetrapropylammonium ($TPrA^+$) | 10 | 11.4 | (20) |
| | Tetrabutylammonium ($TBuA^+$) | 10 | 26.0* | N/A |
| | Tetrapentylammonium ($TPenA^+$) | 10 | N/A | N/A |
| | Tetrahexylammonium ($THexA^+$) | 10 | N/A | N/A |
| Antidiabetics | Metformin | 100 | 1470 | (20) |
| Triptanes | Sumatriptan | 0.5 | 65.9 | (20) |
| | Zolmitriptan | 0.5 | N/A | N/A |
| Tropane alkaloids | Ipratropium | 0.5 | 10.7 | (20) |
| | Trospium | 0.1 | 17.0 | (20) |
| Beta adrenergic agonists | Fenoterol | 0.5 | 0.87 | (20) |
| | Salbutamol | 5 | 395 | (20) |
| | Pirbuterol | 20 | 29,2 | (20) |
| | Orciprenaline | 200 | 530 | (20) |
| | Ractopamine | 1 | 2.17 | (20) |
| | Dobutamine | 5 | 28.4 | (54) |
| Endogenous compounds | Thiamine | 100 | 1057 | (29) |
| | Serotonin | 1 | 663 | (20) |
| Opioids | Methylnaltrexone | 1 | 10.0 | (20) |
| | Norfentanyl | 0.1 | 7.7 | (14) |
| Other | Amisulpride | 3 | 31 | (55) |
| | Ranitidine | 1 | 62.9 | (56) |
| | Zalcitabine | 25 | 1194 | (10) |

N/A not available; * determined in this work

*__Table S4. LC-MS/MS methods used for quantification of intracellular content of individual compounds__ Each LC-MS/MS method (I-IV) allowed the simultaneous quantification of five to seven compounds*

| Method | Analyte | Mass transition (Q1>Q3) | Internal Standard | Mass transition internal standard (Q1>Q3) | Chromatographic separation | Flow rate | Injection volume |
|---|---|---|---|---|---|---|---|
| I | Metformin | 130.1>71.0 | - | - | 0-6 min: 0-13.3% A<br>6-8 min: 13.3% - 44.4% A<br>8-10.5 min: 0% A | 0.55 ml/min | 5 µL |
| | Sumatriptan | 296.3>57.9 | Sumatriptan-d6 | 302.1>64.1 | | | |
| | Zolmitriptan | 288.0>58.0 | | | | | |
| | Zalcitabine | 211.9>112.0 | | | | | |
| | Ipratropium | 332.2>124.2 | Trospium-d8 | 400.1>190.1 | | | |
| | Trospium | 392.1>164.0 | | | | | |
| | Methylnaltrexon | 356.2>338.2 | | | | | |
| II | Fenoterol | 304.5>135.0 | Fenoterol-d6 | 310.3>109.1 | 0-4 min: 2% A<br>4.1-7 min: 15% A<br>7-9 min: 15%-30% A<br>9.1-9.6 min: 80% A<br>9.7-12 min: 2% A | 0.55 ml/min | 5 µL |
| | Salbutamol | 240.5>222.0 | | | | | |
| | Pirbuterol | 241.0>167.2 | | | | | |
| | Orciprenaline | 212.0>194.3 | | | | | |
| | Ractopamine | 302.0>164.0 | | | | | |
| | Dobutamine | 302.2>137.0 | | | | | |
| III | Tetraethylammonium (TEA$^+$) | 130.0>86.0 | TBuA$^+$ | 242.1>142.3 | 0-0.5 min: 30%A<br>0.5-1.5 min: 30-50% A<br>1.5-3 min: 50-85% A<br>3-4.1 min: 85% A<br>4.2-5.5 min: 30 % A | 0.65 ml/min | 3 µL |
| | Tetramethylammonium (TMA$^+$) | 74.2>58.3 | TEA$^+$ | 130.0>86.0 | | | |
| | Benzyl-Triethylammonium (Benzyl-TEA$^+$) | 192.0>90.9 | | | | | |
| | Tetrapropylammonium (TPrA$^+$) | 185.9>114.1 | | | | | |
| | Tetrabutylammonium (TBuA$^+$) | 242.1>142.3 | | | | | |
| | Tetrapentylammonium (TPenA$^+$) | 298.1>170.3 | | | | | |
| | Tetrahexylammonium (THexA$^+$) | 354.5>198.3 | | | | | |
| | Tetraheptylammonium (THepA$^+$) | 410.5>142.3 | | | | | |
| IV | Amisulpride | 370.3>242.0 | Amisulpride-d5 | 375.1>241.9 | 0-2 min: 3% A<br>2-6 min: 3-20% A<br>6-8.1 min: 20% A<br>8.1-9 min: 80% A<br>9-11 min: 3% A | 0.5 ml/min | 5 µL |
| | Ranitidine | 314.9>176.2 | Ranitidine-d6 | 321.2>176.0 | | | |
| | Thiamin | 264.9>122.0 | Thiamin-d3 | 269.1>125.0 | | | |
| | Serotonin | 177.0>160.0 | Serotonin-d4 | 180.8>163.9 | | | |
| | Norfentanyl | 232.8>84.2 | Fenoterol-d6 | 310.3>109.1 | | | |

## References


10. Redeker, K.-E. M., Jensen, O., Gebauer, L., Meyer-Tönnies, M. J., and Brockmöller, J. (2022) Atypical Substrates of the Organic Cation Transporter 1. *Biomolecules* **12**, 1664 10.3390/biom12111664 PMID 36359014
14. Meyer, M. J., Neumann, V. E., Friesacher, H. R., Zdrazil, B., Brockmöller, J., and Tzvetkov, M. V. (2019) Opioids as Substrates and Inhibitors of the Genetically Highly Variable Organic Cation Transporter OCT1. *J Med Chem* **62**, 9890–9905 10.1021/acs.jmedchem.9b01301 PMID 31597043
20. Meyer, M. J., Schreier, P. C. F., Basaran, M., Vlasova, S., Seitz, T., Brockmöller, J., Zdrazil, B., and Tzvetkov, M. V. (2022) Amino acids in transmembrane helix 1 confer major functional differences between human and mouse orthologs of the polyspecific membrane transporter OCT1. *The Journal of biological chemistry* **298**, 101974 10.1016/j.jbc.2022.101974 PMID 35469921
21. Suo, Y., Wright, N. J., Guterres, H., Fedor, J. G., Butay, K. J., Borgnia, M. J., Im, W., and Lee, S.-Y. (2023) Molecular basis of polyspecific drug and xenobiotic recognition by OCT1 and OCT2. *Nature structural & molecular biology* **30**, 1001–1011 10.1038/s41594-023-01017-4 PMID 37291422
23. Zhang, S., Zhu, A., Kong, F., Chen, J., Lan, B., He, G., Gao, K., Cheng, L., Sun, X., Yan, C., Chen, L., and Liu, X. (2024) Structural insights into human organic cation transporter 1 transport and inhibition. *Cell Discov* **10**, 30 10.1038/s41421-024-00664-1 PMID 38485705
29. Meyer, M. J., Tuerkova, A., Römer, S., Wenzel, C., Seitz, T., Gaedcke, J., Oswald, S., Brockmöller, J., Zdrazil, B., and Tzvetkov, M. V. (2020) Differences in Metformin and Thiamine Uptake between Human and Mouse Organic Cation Transporter 1: Structural Determinants and Potential Consequences for Intrahepatic Concentrations. *Drug Metab Dispos* **48**, 1380–1392 10.1124/dmd.120.000170 PMID 33037045
53. Abramson, J., Adler, J., Dunger, J., Evans, R., Green, T., Pritzel, A., Ronneberger, O., Willmore, L., Ballard, A. J., Bambrick, J., Bodenstein, S. W., Evans, D. A., Hung, C.-C., O'Neill, M., Reiman, D., Tunyasuvunakool, K., Wu, Z., Žemgulytė, A., Arvaniti, E., Beattie, C., Bertolli, O., Bridgland, A., Cherepanov, A., Congreve, M., Cowen-Rivers, A. I., Cowie, A., Figurnov, M., Fuchs, F. B., Gladman, H., Jain, R., Khan, Y. A., Low, C. M. R., Perlin, K., Potapenko, A., Savy, P., Singh, S., Stecula, A., Thillaisundaram, A., Tong, C., Yakneen, S., Zhong, E. D., Zielinski, M., Žídek, A., Bapst, V., Kohli, P., Jaderberg, M., Hassabis, D., and Jumper, J. M. (2024) Accurate structure prediction of biomolecular interactions with AlphaFold 3. *Nature* **630**, 493–500 10.1038/s41586-024-07487-w PMID 38718835
54. Jensen, O., Brockmöller, J., and Dücker, C. (2021) Identification of Novel High-Affinity Substrates of OCT1 Using Machine Learning-Guided Virtual Screening and Experimental Validation. *Journal of medicinal chemistry* **64**, 2762–2776 10.1021/acs.jmedchem.0c02047 PMID 33606526
55. Dos Santos Pereira, J. N., Tadjerpisheh, S., Abu Abed, M., Saadatmand, A. R., Weksler, B., Romero, I. A., Couraud, P.-O., Brockmöller, J., and Tzvetkov, M. V. (2014) The poorly membrane permeable antipsychotic drugs amisulpride and sulpiride are substrates of the organic cation transporters from the SLC22 family. *AAPS J* **16**, 1247–1258 10.1208/s12248-014-9649-9 PMID 25155823
56. Meyer, M. J., Seitz, T., Brockmöller, J., and Tzvetkov, M. V. (2017) Effects of genetic polymorphisms on the OCT1 and OCT2-mediated uptake of ranitidine. *PLoS ONE* **12**, e0189521 10.1371/journal.pone.0189521 PMID 29236753
